# Design and Fabrication of New High Entropy Alloys for Evaluating Titanium Replacements in Additive Manufacturing

**DOI:** 10.3390/ma13133001

**Published:** 2020-07-06

**Authors:** Prashant Sarswat, Taylor Smith, Sayan Sarkar, Arun Murali, Michael Free

**Affiliations:** Department of Materials Science and Engineering, University of Utah, Salt Lake City, UT 84112, USA; taylor.james.smith@utah.edu (T.S.); sayan.ju92@gmail.com (S.S.); arun.murali@utah.edu (A.M.)

**Keywords:** additive manufacturing, titanium alloys, high entropy alloys, corrosion, mechanical properties

## Abstract

High entropy alloys (HEAs) were prepared using the powder bed fusion (PBF) technique. Among titanium free alloys AlCoCrFeNiMn, CoCr_1.3_FeMnNi_0.7_, AlCoCrFeNi_1.3_, and AlCoCr_1.3_FeNi_1.3_ have been further investigated. A cost comparison was done for these four alloys as well as the titanium-based alloys AlCoCrFeNiTi and AlCo_0.8_CrFeNiTi. Such a comparison was done in order to evaluate the performance of the titanium-free alloys as the estimated cost of these will be less than for Ti-based HEAs. Hence, we have chosen four titanium free alloys and two titanium-based alloys for further processing. All these alloys were fabricated and subsequently characterized for phase, purity and performance. Scanning electron microscopy-based images were captured for microstructure characterization. EIS-based tests and potentiodynamic scans were performed to evaluate corrosion current. Hardness tests were performed for mechanical properties evaluation. Additional testing using factorial design tests was performed to evaluate the effects of various parameters to create better PBF-based HEA samples. EBSD tests, accelerated corrosion tests (mass loss), chemical analysis after degradation, microstructure analysis before and after degradation, and mechanical property comparison for finalized samples and other similar tests were executed. The details about all these HEAs and subsequent laser processing as well as behavior of these HEAs have been included in this study. It has been observed that some of the selected alloys exhibit good performance compared to Ti-based alloys, especially with respect to improvements in elastic constant and hardness relative to commercially pure Ti.

## 1. Introduction

High entropy alloy fabrication generally utilizes multiple metallic elements in near-equiatomic ratios [1]. The random solid solution of a specific high entropy combination (HEA) has a subjective (arbitrary) distribution of the elements situated along lattice points [1]. Extraordinary properties (such as high ductility and fracture toughness, specific strength, and better mechanical performance at elevated temperature) have been exhibited by various HEAs, which enable their use and expand their potential for a variety of present and anticipated future applications. Based on literature data, seven general families of HEAs (CCAs) have been reported. HEAs are primarily alloys with disordered solid solutions. Solid solution reinforcement is more pronounced for single-phase HEAs than for standard alloy systems (such as stainless steels, bronze, brass, 3xxx, 5xxx, and alpha Ti alloy) [1,2]. In the case of HEAs, the magnitude of hardening of a solid solution is greater than seen in standard binary alloys. The controlled dissemination of auxiliary phases is necessary to achieve adequate mechanical properties at elevated temperature [1]. In most cases, HEAs are synthesized using the arc-melting or spark plasma sintering (SPS) methods, as demonstrated in more than 215 research papers [3]. Other methods are also used to synthesize HEAs or multicomponent systems, including magnetron sputtering, laser-based deposition, powder metallurgy, and splat quenching [3,4,5,6,7,8,9]. In the case of arc melting, comprehensive re-melting and ingot inverting is necessary in order to fabricate a consistent, homogeneous nature alloy. Likewise, it is important to utilize refining and powder alloying when using the SPS technique. Many limitations of form and scale are other considerations when considering the use of these techniques. Thus, is not very likely that any of these advanced synthesis methods will be suitable for industrial and manufacturing routes [10]. Based on the research pattern and current implementation of real-world problems, it can be concluded that future use of HEAs may be limited to highly challenging and specialized applications. The potential advantage of HEAs will alleviate problems including high control levels and inherent complexities [10]. Therefore, techniques which are less complicated, fast, and can provide a high degree of control will be favored in the future [10]. Of these techniques, laser additive manufacturing, which is the focus of our current study, is one of the extensively studied techniques used in modern processing mainly because of its inherent advantages, including the ability to generate high temperatures in a very short time period, fast quenching rate, high design flexibility, the ability to print complex parts in a single run, low lead time, few restrictions, low manufacturing skills, less waste generation, and rapid part modification ability due to the ease of modifying computer-controlled equipment programming [11,12,13,14]. Using an additive manufacturing technique, site-specific microstructure control or alteration is also possible. The combination of HEAs and laser processing is appealing due to a rapid quench rate, thereby reducing the probability of secondary phase formation that happens more frequently with slow cooling and a low associated thermodynamic driving force. The rapid cooling also limits the particle diffusion and nucleation of the various undesirable intermetallic compounds in the alloy that commonly result in brittleness. In the case of metal alloy additive manufacturing, to tie them together, the mixture of metal powders in layers is subjected to high-energy centered sources such as electron beam, high-power laser, or plasma. High-energy beam penetration enables the aggregation, fusion or sintering of metals. Among additive manufacturing techniques powder bed fusion (PBF), commercially known selective laser melting (SLM), is a commonly used technique for the development of HEAs [10,15]. In this case a high-power laser is used to melt and fuse a metallic powder in order to get the desired material and shape. Details about the PBF technique and its associated use have been discussed in previous published results [16,17].

The last few years have seen great improvements in the technology solutions of additive manufacturing (AM) which result in the production of fully functional parts using titanium and its alloys. Although powder bed fusion technologies offer the capacity to create hollow near net forms with greater resolution, concentrated energy-based technologies provide the ability to attach features to existing components as well as repair/remanufacturing of the damaged parts. Most studies show that AM materials’ mechanical properties and durability are as good or even better than those of conventionally manufactured titanium alloys. Choosing the right AM technology along with appropriate design optimization will lead to very substantial savings through vastly reduced buy-to-fly ratios, weight reduction overall and scrap reduction. A variety of Ti additive manufactured parts have been produced using spherical gas atomized or plasma rotating electrode processes. The powder cost can be ~10% of the overall cost of the component [18,19,20].

Additive manufacturing has tremendous potential for producing a variety of titanium parts for many aerospace applications. Two of the main challenges for additive manufacturing (AM) of titanium parts are the cost of the titanium powder and the fatigue performance of the final parts. Many of the proposed replacement alloys for titanium have titanium as one of the alloying elements, and most of the other potential replacement alloys use one or more expensive elements, making the parts cost prohibitive. Other lower cost replacement alloys do not have the necessary corrosion resistance or performance properties to adequately replace titanium. Thus, there is a need to find and evaluate other alloys that meet the cost and performance requirements of the additively manufactured parts needed for aerospace applications. The proposed research addresses these needs and opportunities using low cost, high entropy alloys (HEAs) and compositionally complex (often multiphase) alloys (CCAs) and targeted control of the additive manufacturing processing parameters. This study utilized first principles calculations and cost analysis to guide the development of low cost HEA/CCAs with excellent strength and corrosion resistance as well as moderate density. The information will be utilized for the proposed alloys that exhibit excellent performance as indicated by first principles calculations.

This research involved shortlisting the key elements that are being used to create corrosion resistant alloys. Some of the selected elements are Al, Mo, Cr, Ni, Mn, and other passive metals such as Ti [21]. The addition of the titanium has been done to evaluate the comparative performance of alloys without titanium. In the case of equivalent or better performance, further research can be done for development of Ti-free HEAs. The addition of Cr has a tendency to make the proposed alloys ‘stainless’ as it tends to form a protective chromium oxide-based passive film. Similarly, Mo often provides passivation due to the formation of a protective MoO_2_-based passive film, or it can generate MoO_4_^2−^ species. Note that in these alloys, the optimum content of each constituent is important. One such example is Al_x_CoCrFeNi alloys, where alloys with >5 wt.% Al exposed to H_2_SO_4_ solutions exhibited higher mass loss than alloys with lower Al content [22]. Note that there are some equiatomic alloys that utilize many of these constituent elements, however no information is available in the literature regarding HEAs composed of different ratios of these constituents. Hence, enthalpy, entropy, elastic constants, and other related parameters of stability were evaluated for selected alloys. The relative cost of fabrication of proposed HEAs was also calculated. Most competitive alloys (based on preliminary estimation) will be further investigated by varying the ratio of key elements and new properties will be noted. DFT simulations were conducted using first principles-based methodologies by implementing the general gradient approximation (GGA) with Perdew-Burke-Ernzerhof (PBE) exchange-correlation functional [23]. High performance, less expensive alloys were developed. These alloys will be synthesized and further explored using an additive manufacturing technique.

## 2. Materials and Methods

### 2.1. Cost Analysis of Selected HEAs

In order to analyze the cost of fabrications, the following factors were considered:

- Cost of the metal powder (size <100 micrometer, usually atomized mill product)
- Cost of labor (metal mixture preparation efforts for selective laser melting)
- Cost of laser melting (equipment cost, hourly charges; laser power used in melting)
- Cost of post-deposition sample preparation steps (removal, polishing etc.)

**Figure 1:**
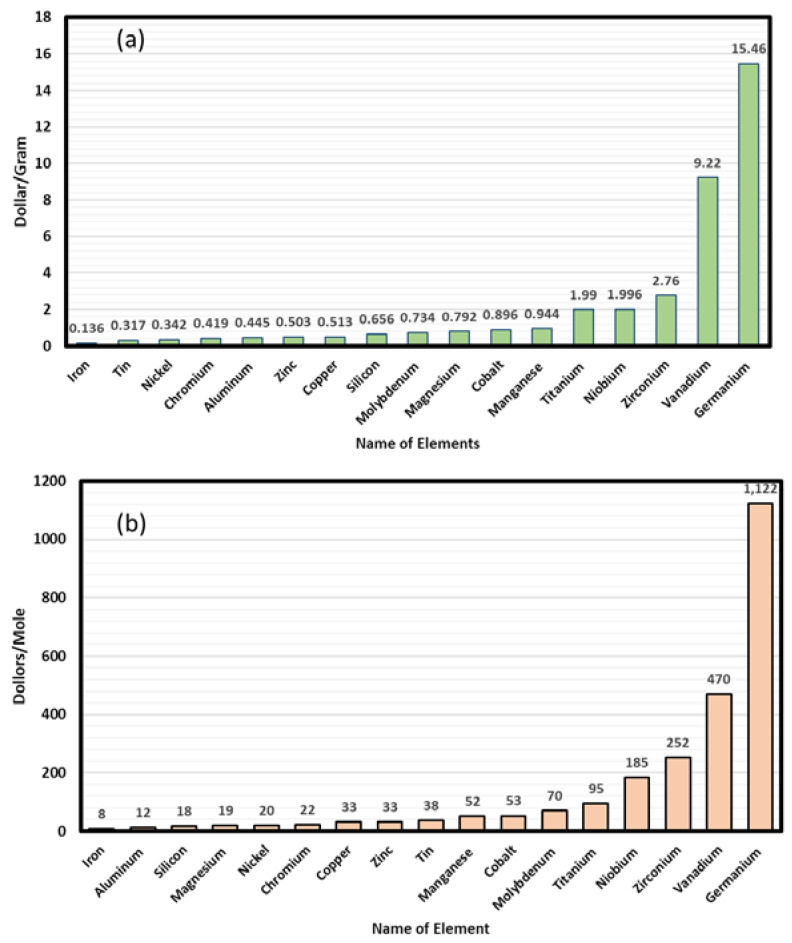
(**a**) Cost/gram of different metal powder to be used for additive manufacturing. (**b**) Cost/mole of different metal powder to be used for additive manufacturing.

In the present case, the assumption that cost of labor, laser melting, post deposition sample cleaning are similar for each HEA, hence, for the present case, the deciding factor for cost will be the cost of the constituent metallic powders. Various databases and metal supplier cost lists have been examined (e.g., Sigma Aldrich and Alfa Aesar) for cost comparison. Initially the authors have identified ~67 HEAs from the literature (see Table A1) that have been synthesized or simulated [24]. These HEAs were shortlisted from various alloy systems and compositions based on facts that they are mainly solid solution (SS) type compositions with negligible (or nil) intermetallic (IM) compound formation. Another restriction was that alloy must be single phased. Although combinations of phases such as FCC + B2, BCC + FCC, FCC + L1_2_, BCC + 2FCC + B2 + σ, BCC + FCC + C15 + L2_1_, BCC + B2 + σ, BCC + FCC have been often observed, most of the shortlisted HEAs are either FCC, BCC, or HCP type. Some alloys (MoPdRhRu and MoRhRu) have been excluded due to their use of expensive REEs or other precious metals. In most of the HEAs, the commonly used elements are Fe, Al, Si, Mg, Ni, Cr, Cu, Zn, Sn, Mn, Co, Mo, and Ti. Other frequently used elements are Ge, Zr, and V, but the cost of these elements is higher than for other metals. Germanium is eight times more expensive than titanium, whereas vanadium is four times more expensive than titanium. Hence, in design of new HEAs, these elements have been excluded. The average/estimated price of the elements used in HEAs is shown in Figure 1a,b. In the list of elements (excluding Nb, Zr, V, and Ge), titanium is the most expensive, followed by Mn, Co, Mg, and Si. Fe, Sn, Ni, Cr, and Al are the least expensive metals in this list. Thus, it is easy to understand that the cost of HEAs will vary according to the choice of constituents.

**Figure A1:**
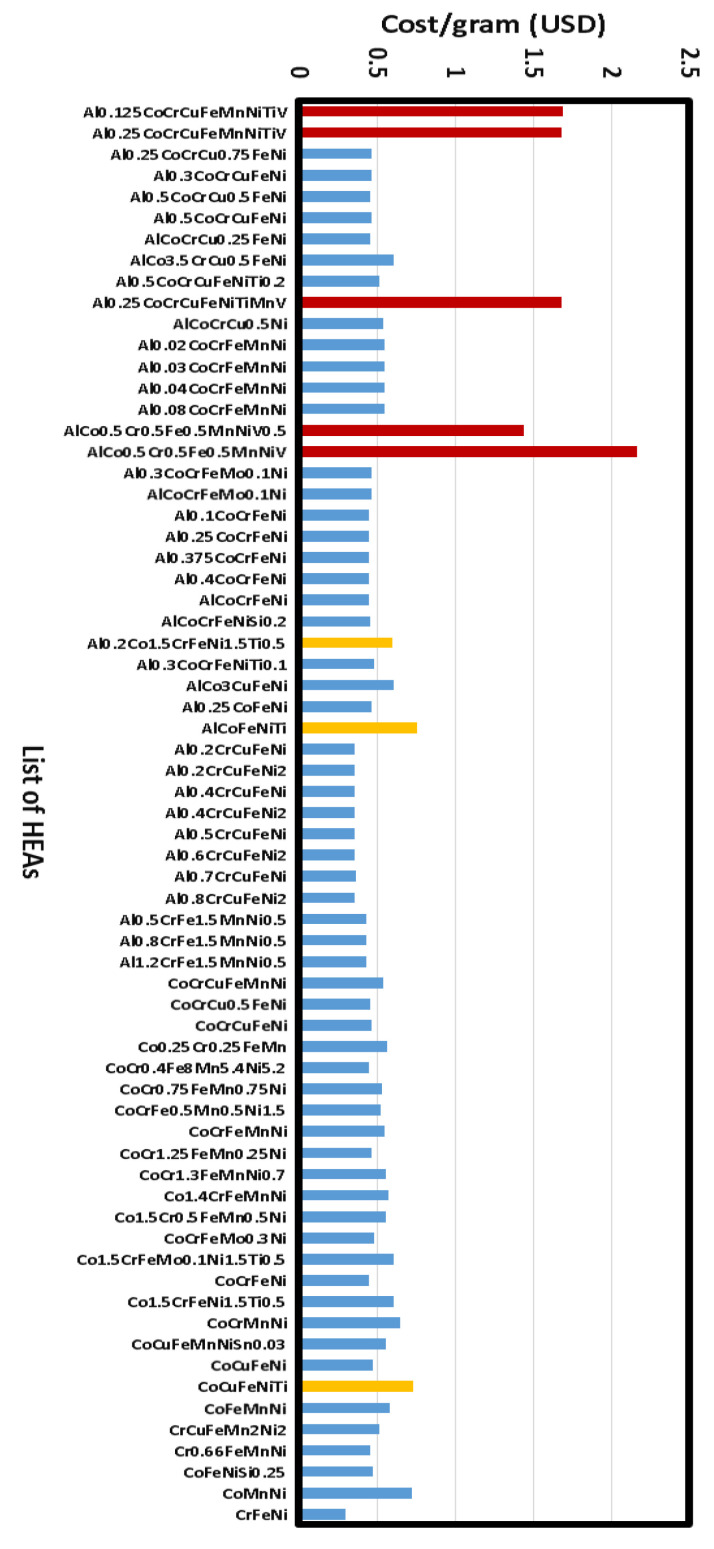
Estimated cost/gram of different HEAs to be used for additive manufacturing. Red color bars indicate very expensive HEAs whereas yellow bars indicate alloys with Ti.

**Figure A2:**
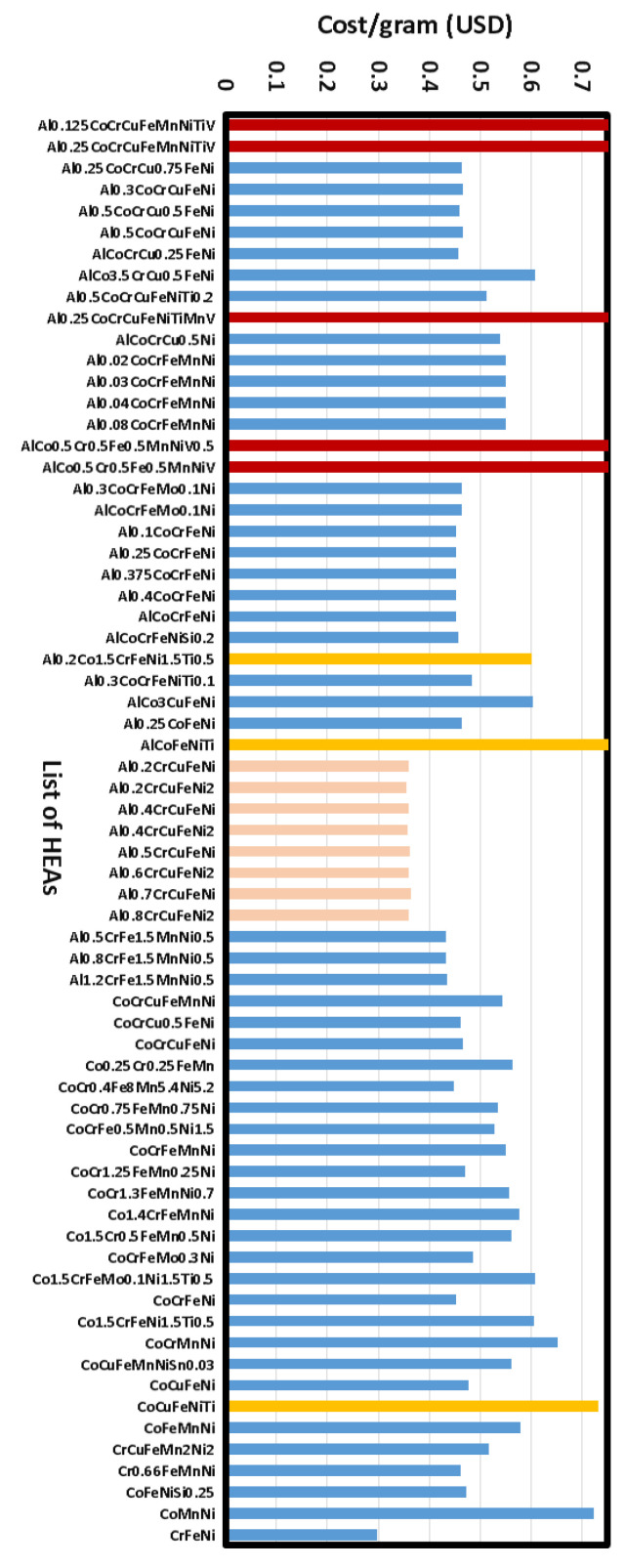
Estimated cost/gram of different HEAs to be used for additive manufacturing. Red color bars indicate very expensive HEAs whereas yellow bars indicate alloys with Ti. The least expensive alloys are shown in pink bars.

The authors have identified the most expensive HEAs (shown in red bars), moderately expensive HEAs (shown in orange bars), and the least expensive HEAs (pink bars) have been identified as shown in Figure A1 and Figure A2. At this point, the selection of alloys should be based on not only cost, but according to the needs and desired properties. It was observed that the least expensive HEAs in the graphs are Cu-based alloys (Al_0.2_CrCuFeNi, Al_0.2_CrCuFeNi_2_, Al_0.4_CrCuFeNi, Al_0.4_CrCuFeNi_2_, Al_0.5_CrCuFeNi, Al_0.6_CrCuFeNi_2_, Al_0.7_CrCuFeNi, Al_0.8_CrCuFeNi_2_). It has been observed that Cu-based CCAs showed enhanced cathodic kinetics relative to Cu-free CCAs of similar compositions. The mass loss rate was also ordered according to the ranking FeCoNiCrCu > FeCoNiCrCu_0.5_ > FeCoNiCr. In the case of immersion testing for mass loss (3.5% NaCl) a number of small pits were observed on the surface of the Cu-free FeCoNiCr alloy; however, in the Cu-based alloys the interdendritic phase was preferentially dissolved. From several reports, it appears that Cu is detrimental to the corrosion resistance of HEAs as Cu enhances elemental segregation, forming a Cu-rich interdendritic phase and a Cu-lean (and Cr-rich in Cr-containing CCAs) dendritic phase. Hence, Cu-based CCAs were not deeply explored in this report, though some thermodynamic calculations have been shown. However, it was noted that HEAs containing the constituent elements Al, Co, Fe, Ni, Mn, and Cr are more expensive than Cu-based HEAs but less expensive than other shortlisted alloys, as shown in Figure A1 and Figure A2. Hence, additional calculations were conducted for these alloys. Note that titanium of the same size and availability is, for purposes of this comparison, about $2/gram. This is higher than bulk purchases, but in order to compare it fairly with other powdered metals, current small quantity purchase prices were used.

### 2.2. Free Energy, Enthalpy, and Entropy Calculations

Free energies of high entropy alloys, expressed as a function of *F*(*V*,*T*) are indicators of driving force required to form a single phase like B2_BCC (e.g., AlCoCrFeNi). The driving force required to form a single phase HEA can be described by using an adiabatic approximation as below:

(1) $$F\left(V,T\right)=E_{static}\left(V\right)+F_{vib\;}\left(V,T\right)+F_{elec\;}\left(V,T\right)-TS_{config}\left(T\right)$$

where, $E_{static}$ refers to the static energy at 0 K. $F_{vib}$ refers to the vibrational free energy and can be predicted with the help of Debye–Gruneisen model [25]. In order to calculate electronic free energy, Sommerfeld’s model [26] of independent free electrons is used:

(2) $$F_{elec\;}\left(V,T\right)=E_{elec}\;\left(V,T\right)-TS_{elec\;}\left(V,T\right)$$

where, $E_{elec}$ and $S_{elec\;}$refer to the electronic energy and electronic entropy, respectively. The configurational entropy $S_{config}$ for a disordered ideal solution is given by:

(3) $$S_{config}=-R\sum_{i}c_{i}lnc_{i}$$

Enthalpy of mixing is another indicator for the formation of single phase disordered solid solution, which can be given as:

(4) $$\Delta H_{mix}=\overset{N}{\underset{i=1,i\neq j}{\sum }}\omega_{ij}c_{i}c_{j}$$

where, $\omega_{ij}$ = 4 $\Delta H_{A,B}^{mix}$ is the regular interaction parameter between the *i^th^* and *j^th^* elements. CALPHAD calculations helped to predict these thermodynamic parameters which are essential for analyzing the phase stability of these single phase HEAs.

### 2.3. Calculations of Elastic Constants

Energy-strain and stress-strain methods are commonly used to calculate the elastic properties of materials. Cell optimization (both cell vectors and atomic positions are optimized) was first carried out to obtain the approximate lattice constant. In order to calculate equilibrium lattice constant a_0_, equilibrium volume *V*_0_ and bulk modulus *B*, five different volume at 0.98a, 0.99a, a, 1.01a, and 1.02 were constructed for geometry optimization (atomic positions are optimized). *V*_0_ and *B* were obtained by fitting the first-principles calculated total energies versus volume curve with Birch-Murnaghan equation of state, namely [27,28]:

(5) E(V)=E0+9V0B16{[(V0V)23−1]3B′+[(V0V)23−1]2[6−4(V0V)23]1}

In these first principles-based DFT simulations the framework of the norm-conserving pseudopotential was utilized. This exchange-relation was used for density of states (DOS) calculations as well as for the total energy of the system for all of the proposed HEAs. In order to solve Kohn-Sham equations for electronic properties simulations and structure optimization, the plane wave pseudopotential technique was used in the QUANTUM-ESPRESSO2 package. For the procedure utilized in designing new HEAs, 6 × 1 × 1 supercells of the unit cell or 5 × 1 × 1 supercell, were developed keeping the rule that all components would distribute haphazardly in the periodic model. The cut-off condition of the periodic crystals would be determined by the maximum separation distance between same-element atoms A limited memory Broyden-Fletcher-Goldfarb-Shanno (LBFGS) algorithm was implemented for structural optimization of the various configurations of the HEAs supercells and the total energy was used in the PBE exchange–functional correlation that was also utilized to measure the energy/atom formation. After structural and geometry optimization, these alloys were observed to crystallize in a FCC structure.

A series of small strains were applied to equilibrium lattice configuration to get elastic constants. In order to keep the elastic behavior of crystals, the applied strains should be relatively small, so the strains adopted are *ε* = −0.02, −0.01, +0.01, and +0.02. Cubic crystals have three independent elastic constants *C*_11_, *C*_12_ and *C*_44_, and they can be obtained by fitting strains against the corresponding total energies. The following strain matrix was used [27,28]:

(6) $$\left(\begin{array}{ccc} 1+\sigma & 0 & 0\\ 0 & 1 & 0\\ 0 & 0 & 1 \end{array}\right),\left(\begin{array}{ccc} 1+\sigma & 0 & 0\\ 0 & 1+\sigma & 0\\ 0 & 0 & 1 \end{array}\right)\;and\;\left(\begin{array}{ccc} 1 & \frac{\sigma }{2} & \frac{\sigma }{2}\\ \frac{\sigma }{2} & 1 & \frac{\sigma }{2}\\ \frac{\sigma }{2} & \frac{\sigma }{2} & 1 \end{array}\right)$$

which are related to the following energy changes:

(7) $$E\left(\sigma \right)=E\left(0\right)+\frac{1}{2}C_{11}V_{0}\sigma^{2}$$

(8) $$E\left(\sigma \right)=E\left(0\right)+(C_{11}+C_{22})V_{0}\sigma^{2}$$

(9) $$E\left(\sigma \right)=E\left(0\right)+\frac{3}{2}C_{44}V_{0}\sigma^{2}$$

The total energy vs. strain values for different alloy systems are shown below and the obtained elastic constants were calculated from different relationships. The shear modulus (‘*G*’) was then evaluated from the elastic constants as:

(10) $$G=\frac{3C_{44}+C_{11}-C_{12}}{5}$$

The Young’s modulus (*E*) is linked to bulk and shear modulus as:

(11) $$E=\frac{9BG}{\left(3B+G\right)}$$

and the Poisson’s ratio is related as:

(12) $$\nu =\frac{3B-2G}{2\left(3B+G\right)}$$

Overall, titanium free high performance HEAs were identified using DFT-based calculations. Initial assessment identified four alloys for further investigation. These alloys were used as a starting point and additional calculations for these sets of alloys were done. In the experimental stage of research actual behavior of these alloys was investigated and any change to improve the properties without significant change in cost of fabrication, can be implemented.

### 2.4. Laser Components

**Figure 2:**
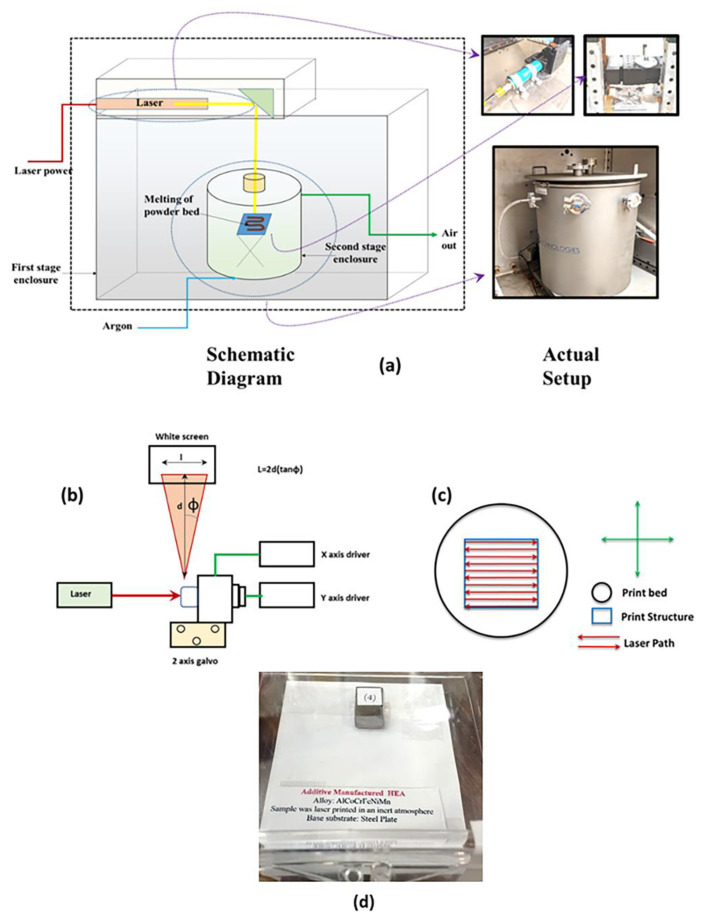
(**a**) A schematic diagram of selective laser melting system used for HEAs synthesis. It can be seen that a two-stage enclosure was used for better safety. (**b**) A schematic diagram of the dual axis mirror system; and (**c**) A schematic diagram of the laser path, print bed, and final print structure; (**d**) a final HEA sample printed using the PBF technique.

For this process, a YLR-500-AC-Y14 500 W ytterbium fiber laser (IPG Photonics, Oxford, MA, USA) with a wavelength of 1064 nm was used in sequence with an IPG collimator to form a 6 mm collimated beam (See Figure 2). The collimated beam then passed through a GVS312 large beam dual axis galvanometer (Thorlabs, Newton, NJ, USA) equipped with a Thorlabs F-Theta scan lens with a focal length of 10 inches.

### 2.5. Galvanometer Setup and Detail of Phidgets Hub for Laser-PBF Processing Control

The galvanometer was connected to a Phidgets Hub and two Phidgets 16-bit Voltage Output DAC cards (Phidgets Inc., Calgary, AB, Canada). This enables the galvanometer system to receive signals ranging from −10 V to 10 V, that can be converted into an optical scan angle ranging from ±23° for the X axis, and +40°, −35° for the Y axis. A communication with the Phidget Hub and DAC card was performed using Microsoft Visual Studio 2019 (Microsoft Corporation, Redmond, WA, USA) with Python code.

The base of the code that allowed for communication between the Phidget devices and the computer was written using existing Phidget code, however, the code responsible for the movements was performed using various for and while loops, which were adjusted to optimize the scan speed and shape of the print bed by adjustment of different input voltage values throughout the code. As voltages were adjusted within the Python code, the step size could be increased or decreased, as well as increasing or decreasing the scan speed. Similarly, by changing other variables within the program, the voltages correlating to print size can be varied. The program was written in such a way that the laser would create a rectangle by starting in the bottom right hand corner and raster back and forth in the X direction, moving up in the Y direction one step as it reached the edges in the X direction. By varying the step sizes, the speed of the raster either increases with larger step sizes or decreases with smaller step sizes. Hatch spacing is then altered by varying the input voltage for the *y*-axis. The sample was rotated to 90° in cases when thicker samples or more layers were needed. The thickness of each layer was around ~0.3 mm.

### 2.6. Optimization of Parameters

For the preliminary runs, pure metal powder (such as Ni) was used in order to preserve the more costly powders. In addition, a powder hopper system installation was used. Steel substrates were used for metal printing. In these substrates, adhesion to the substrate greatly improved, and surface porosity and stress cracking greatly decreased. Scan speed, power input, and layer thickness were all varied until a good first layer was produced. After the first layer melted well enough, subsequent layers were added and a test specimen for one of the six HEA alloys made was thick enough and melted enough to perform testing on it. Once the parameters for one alloy were optimized, the parameters for the remaining five alloys were also optimized. In total, roughly 100 test runs were performed, of which various tests had multiple scans performed. It was noted the power must be at least 29%, or 145 W, in order to get a good melt. Nevertheless, power as high as 32–60%, was used and acceptable samples were created. The scan speed was varied not in measurement of mm/sec but rather in voltage input changed. With smaller voltages input, the step size would be smaller which then results in slower scan speeds. The final scan speed ended up being 0.003 V steps along the X axis and 0.01 V in the Y axis, with a print bed size of just under 2 cm in the Y direction and 0.75 cm in the X direction. The average bed height was ~300 µm.

### 2.7. Details about Closed Atmosphere Tests

A data feedthrough was used that contains a USB feedthrough to control all electrically powered equipment within the chamber for controlled atmosphere tests. The power chords for the powder hopper and z-stage were spliced and connected using MOLEX pins to the thick copper rods in the power feedthrough. The powder hopper needed to be able to move forward and backward on command once the laser was turned off to avoid being melted by the laser. Additionally, it needed to be able to hold enough powder for an entire run while still being light enough to easily glide back and forth over the powder bed. Using a DVD drive as a linear motor, two nuts were mounted onto the front of the stage. The holes in the nuts served as the powder bin. The bolts already have a polished face and slide with ease across the steel substrate and disperse the powder evenly and smoothly. Using a guide laser built into the IPG laser, the python program was executed and tested several times to verify correct placement of the substrate with respect to the print pattern.

Tests were initiated by placing the sample on the sample holder and powder in the hopper and the chamber was sealed. Vacuum pressure was applied for 10 min using a rotary vane vacuum pump with a max capability of 10^−3^ torr. The pump was then turned off and ultra-high purity (UHP) argon was introduced into the chamber. This process was repeated three times to ensure the chamber has been properly evacuated of oxygen and other contaminants. This process was finally optimized in order to create a solid sample without pores, defects, or oxidation.

### 2.8. Morphology, Elemental Characterizations, and Electron Backscatter Diffraction

The microstructures of the six HEA samples were examined using an NovaNano scanning electron microscope (SEM) (Lincoln, NE, USA) equipped with a field emission gun. For our set of measurements, the voltage was 30 kV. To visualize the elemental distribution consistency and precipitation, the elemental spectra and maps of the HEA tests were collected utilizing an energy dispersive X-beam spectroscopy (EDS) detector (EDAX, Lincoln, NE, USA) and in this way, the EDAX Genesis programming could be applied to process the EDS results [29].

### 2.9. Corrosion Behavior of HEAs

On account of corrosion, the exposed sample experiences an electrochemical procedure that causes change or corruption of material essentially into their oxides, dissolved salts (in their ionic form) or hydroxides. For this situation, two half-cell reactions (one anodic and another cathodic response) happen and free electrons in response devoured by other reduction processes. The overall rate of reaction can be determined by ascertaining the difference between the forward and the backward rates, that is proficiently broke down utilizing electrochemical kinetics. It is essential to take note of that the joined reaction rate will be for the most part represented by the rate-determining reaction rate when the half-cell reactions are consolidated, in this condition the total of cathodic currents is equivalent to the sum of anodic currents:

(13) $$\sum \;I_{anodic}=-\sum \;I_{cathodic}$$

Conversely, should irregularity occur, the total effective current in an electrochemical cell can be determined by deducting the cathodic reaction rate from the anodic rate. The Butler-Volmer Equation can be used for this situation to calculate the rate [23]:

(14) $$i=i_{o}\left[\exp\left(\frac{\alpha F\left(\eta \right)}{RT}\right)-\exp\left(\frac{\left(1-\alpha \right)F\left(\eta \right)}{RT}\right)\right]$$

Here α represents the charge transfer coefficient, *η* is the overpotential (*E-E_eq_*), *F* is Faraday steady, *R* denotes the universal gas consistent, *T* is the absolute temperature, and *i*_o_ represents the exchange current density. In most systems *η* is positive for anodic reactions and negative for cathodic reactions. For the planned corrosion and material dissolution rate estimations, electrochemical polarization tests have been performed for all samples at the suitable scan rate. The shape of the Butler-Volmer curve speaks to the strength of the exponential term that controls either the anodic or cathodic reaction. When the overpotential is enormous, either the principal term or second term in condition (14) dominate. Additionally, in the event that *α* = 0.5, the gradients of the cathodic and anodic branches become equivalent; however, this is definitely not a general case. The situation at high overpotential, when overpotential is linearly related with log |*i*|, is utilized to evaluate the Tafel slope and the related corrosion rate. After the recognizable proof of linear Tafel region away from the corrosion potential, the crossing point of extrapolated cathodic and anodic current lines gives the natural reaction potential. The corrosion tests (cyclic potentiodynamic polarizations) were performed using 3.5 wt.% NaCl solution in water [30]. These tests were performed based on the guidelines of the ASTM G61 standard. All the polarization tests were carried out with reference to a saturated calomel electrode (SCE). The counter electrode used in the potentiodynamic polarization tests was platinum. The scan rate employed was 0.2 mV/s during the polarization test. Electrochemical Impedance Spectroscopy (EIS) tests were conducted in the potentiostatic mode with the following parameters: an AC signal amplitude of 5 mV, 5 points/decade and with a wide frequency range of 100 mHz–100 kHz [31]. A Reference 600 potentiostat (Warminster, PA, USA) and a three-electrode system (using platinum as the counter electrode and saturated calomel electrode as a reference) was used during the impedance measurement tests [32,33]. All the results were analyzed using the Electrochem Analyst software (Gamry, Warminster, USA) by considering an equivalent electrical circuit to define the initial approximations of the circuit elements magnitude.

#### Accelerated Immersion Tests

In the stage of corrosion, the material experiences an electrochemical effect that causes change or degradation of material, fundamentally into their oxides, aqueous salts or hydroxides. Accelerated immersion tests were conducted based on ASTM standard G34-0, before the HEA samples were subjected to an elevated temperature corrosive atmosphere. The corrosive solution used for these tests consisted of a mixture of 4 M NaCl, 0.5 M KNO_3_ and 0.1 M HNO_3_. The samples were kept in the corrosive solution for 5 days. All the elements in the HEAs composition were analyzed using an ICP-MS instrument (NexION 1000 ICP-MS, PerkinElmer, Waltham, MA, USA) located at the University of Utah after diluting the solution by a factor of 10.

### 2.10. Hardness Tests

The load applied on the material, the average diagonal of the indentation, and the measured hardness values are shown in Figure A15. The hardness or HV number is calculated with Equation (15).

(15) $$HV=\frac{F}{A}\approx \frac{1.8544\;F}{d^{2}}\left[\frac{kgf}{mm^{2}}\right]$$

where, *H* is the Vickers Pyramid Number, *F* is the load applied in kgf, *A* is the surface area in mm^2^, and d is the average length of the diagonal left by the indenter in mm.

### 2.11 Factorial Design of Experiments Testing

Factorial design of experiments was used to efficiently optimize all parameters and establish the right protocol for HEA laser printing. The dimensions of the samples were ~1 cm × 1 cm × 1 cm for most of the tests, whereas for corrosion tests smaller samples were created. Eight tests were executed for the design of experiment matrix. Factorial design of experiments allows us to study the effect of each factor on the response variable, as well as effects of interactions between individual factors on the response variable. Factors chosen in this case were: effect of individual components (laser power, scan speed, and thickness of substrate). In most of the factorial experiments each factor has only two levels (high and low). This experimental matrix included 3 factors A (laser power), B (scan speed), and C (thickness) with 2 levels (high and low). The substrate thicknesses were 2 mm (low) and 4 mm (high). The laser power levels of 30% (of total maximum for high or 145 W) and 25% (of total maximum for low~130 W) were evaluated. The scan speed was also a variable (in term of 5 mV and 3 mV applied bias). The selected highest and lowest values of each factor were determined from their actual range of variation, literature data, as well as some preliminary trial runs (single factor study) that were conducted so as to guide the actual process effectively. This resulted in eight treatment combinations. A set of tests that follow the factorial design approach with variables A, B and C tested at high (+) and low (−) levels is presented in Table A2. The value of main effect parameter and interaction effect parameter is indicative of how strong individual factors affect the response variable. The null outcome indicates the neutrality of the parameter effect. The relative effects of the parameters are shown below: The test results were analyzed using a statistical analysis software (Minitab-17, PA, USA) where effects of each parameter was investigated. After analysis different charts and graphs were created. A Pareto chart of effects, interaction plot for response, main effect plots for response, and contour and surface charts of responses is shown.

(A) = (−Y1 + Y2 −Y3 + Y4¬ − Y5 + Y6 −Y7 + Y8)/8(16)

(B) = (−Y1 − Y2 +Y3 + Y4¬ − Y5 −Y6 +Y7 + Y8)/8(17)

(C) = (−Y1 − Y2 − Y3 − Y4¬ + Y5 + Y6 + Y7 + Y8)/8(18)

(AB) = (Y1 + Y2 − Y3 − Y4¬ − Y5 − Y6 + Y7 + Y8)/8(19)

(AC) = (Y1 − Y2 + Y3 − Y4¬ − Y5 − Y6 − Y7 + Y8)/8(20)

(BC) = (Y1 + Y2 − Y3 − Y4¬ − Y5 − Y6 + Y7 + Y8)/8(21)

(ABC) = (−Y1 + Y2 + Y3 −Y4¬ + Y5 − Y6 − Y7 + Y8)/8(22)

Mean = (Y1 + Y2 + Y3 + Y4¬ +Y5 + Y6 + Y7 + Y8)/8(23)

## 3. Results and Discussion

### 3.1. Elastic Properties

**Figure 3:**
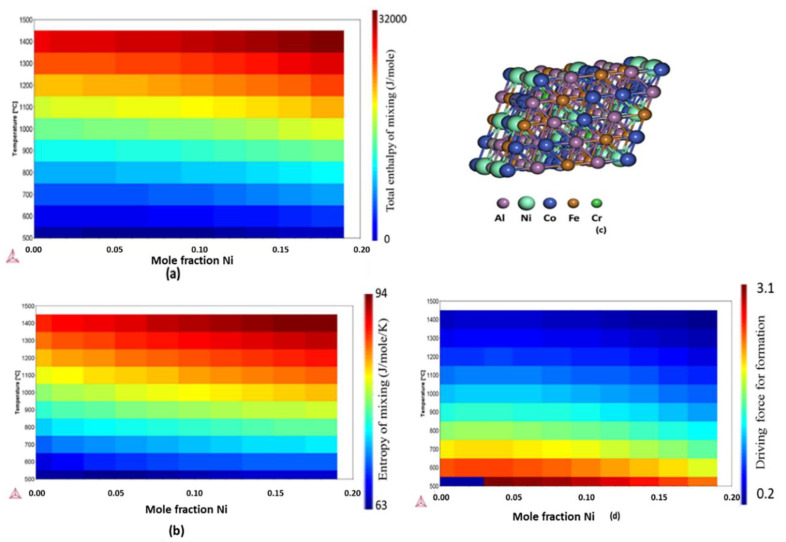
Role of Ni on the thermodynamic stability of HEA AlCoCrFeNi (**a**) Total enthalpy of mixing (J/mole) (**b**) Entropy of mixing (J/mole/K) (**c**) Optimized BCC lattice (**d**) Driving force for formation of B2_BCC per mole of AlCoCrFeNi.

**Figure A3:**
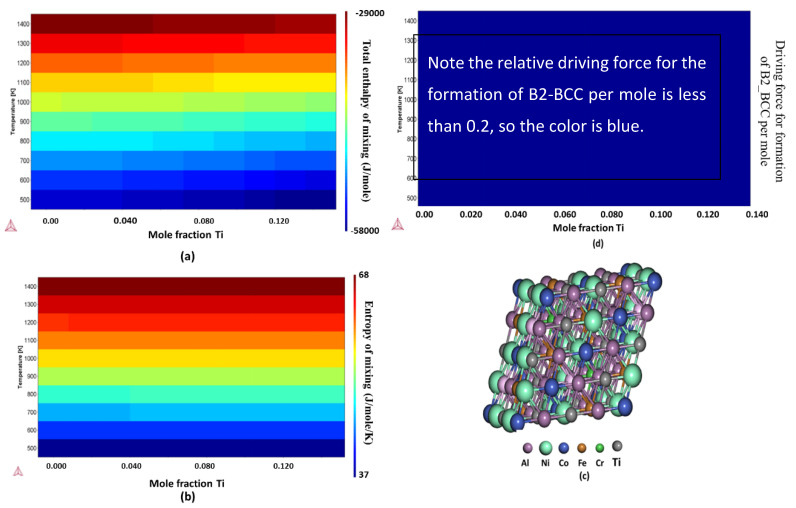
Role of Ti on the thermodynamic stability of HEA AlCoCrFeNiTi (**a**) Total enthalpy of mixing (J/mole) (**b**) Entropy of mixing (J/mole/K) (**c**) Optimized BCC lattice (**d**) Driving force for formation of B2_BCC per mole of AlCoCrFeNiTi.

Figure 3 shows the effect of Ni on the thermodynamic stability of HEA AlCoCrFeNi. It was noted that the equimolar Ni addition dominates the entropy of mixing over enthalpy of mixing. It was observed that Ni addition increases the driving force to form BCC_B2 phase of AlCoCrFeNi as the content of Ni is increased at temperatures below 1000 °C, however at temperatures over 1000 °C, increasing Ni addition has negligible effect on the driving force required to form the B2_BCC phase. The Figure A3 shows the effect of Ti on the thermodynamic stability of AlCoCrFeNiTi, and the addition of Ti increases both entropy of mixing and enthalpy of mixing, such that there is no significant effect on the driving forced required to form B2_BCC phase.

(24) $$\Omega =\frac{T_{m}\times \Delta S_{mix}}{\left|\Delta H_{mix}\right|}$$

**Figure 4:**
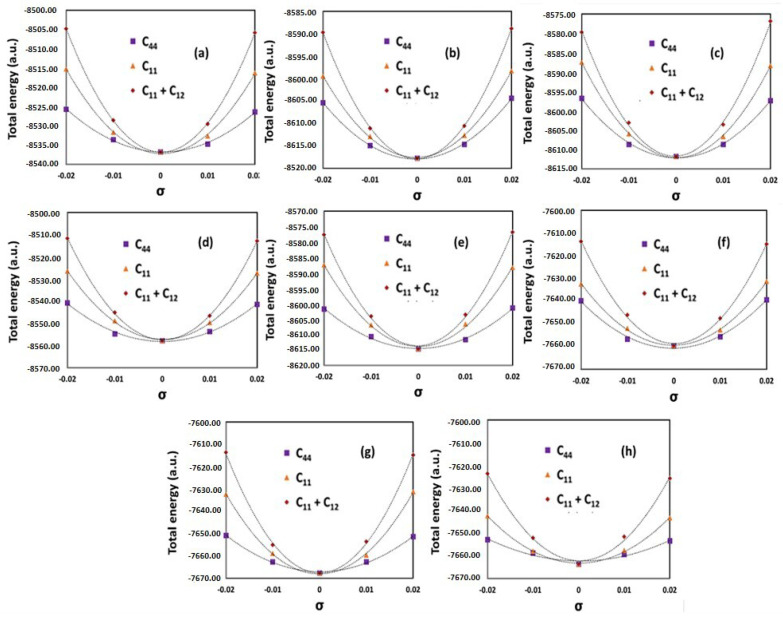
Plots showing energy changes due to application of strains (deformation) and its impact on the elastics constants *C*_44_, *C*_11_ & *C*_11_ + *C*_22_ for (**a**) AlCoCr_1.3_FeNi_1.3_ (**b**) AlCoCrFeNi_1.3_ (**c**) AlCoCrFeNiTi_0.9_ (**d**) AlCo0_.8_CrFeNiTi (**e**) AlCo_0.9_CrFeNiMn_0.9_ (**f**) AlCoFeNiZn_0.85_ (**g**) AlCrFeNiMo_0.1_ (**h**) AlCoCrFeMo_0.1_.

**Figure A4:**
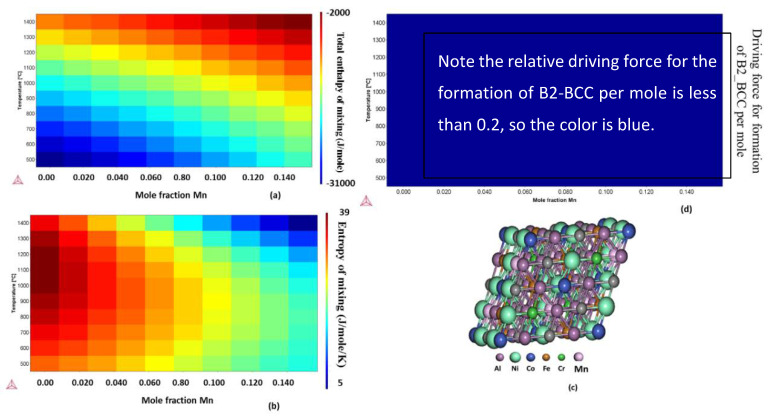
Role of Mn on the thermodynamic stability of HEA AlCoCrFeNiMn (**a**) Total enthalpy of mixing (J/mole) (**b**) Entropy of mixing (J/mole/K) (**c**) Optimized BCC lattice (**d**) Driving force for formation of B2_BCC per mole of AlCoCrFeNiMn.

**Figure A5:**
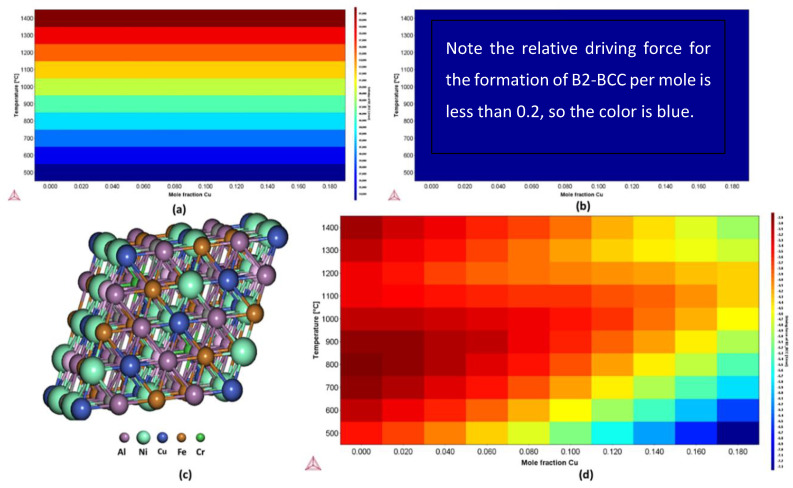
Role of Cu on the thermodynamic stability of HEA AlCuCrFeNi (**a**) Total enthalpy of mixing (J/mole) (**b**) Entropy of mixing (J/mole/K) (**c**) Optimized BCC lattice (**d**) Driving force for formation of B2_BCC per mole.

**Figure A6:**
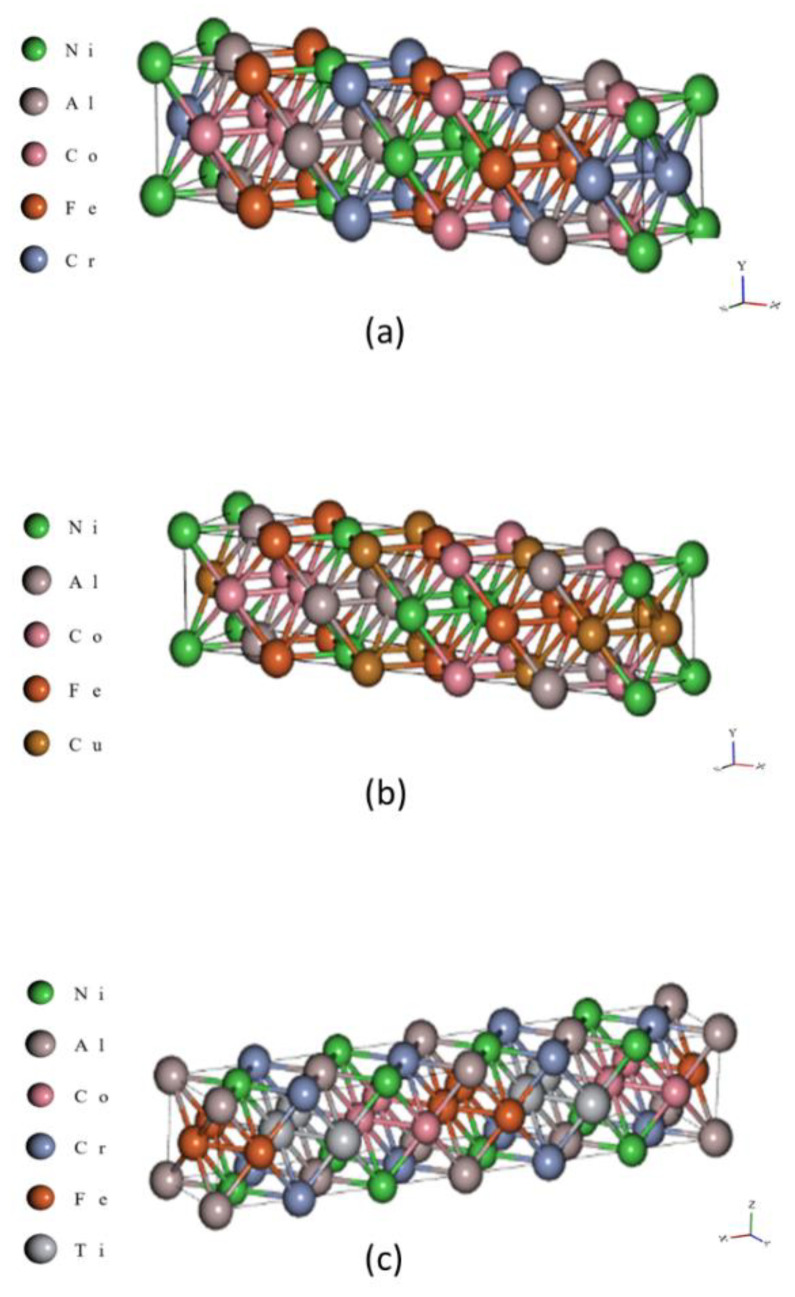
Deformed HEA geometries for the calculation of elastic constants in (**a**) AlCoCrFeNi, (**b**) AlCoCuFeNi and (**c**) AlCoCrFeNiTi.

**Figure A7:**
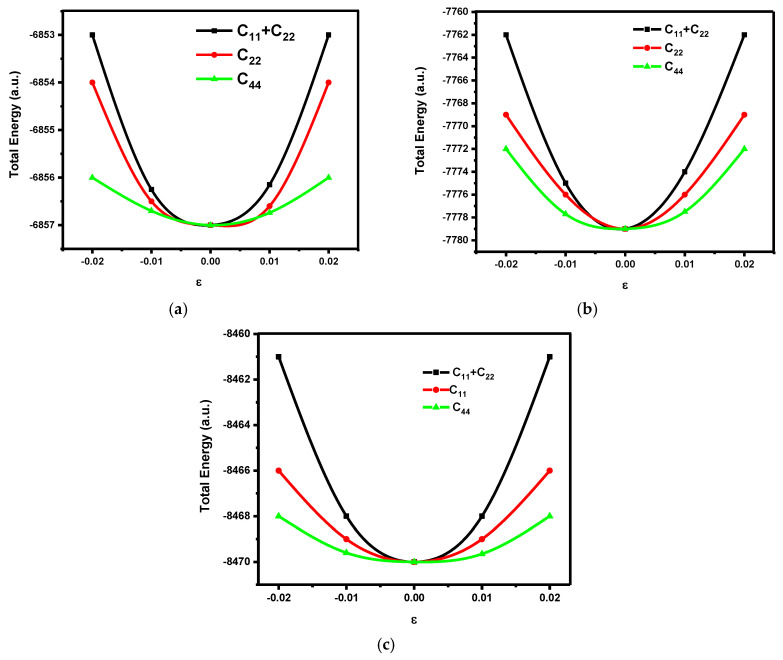
Energy variations due to application of strains (deformation) and its influence on the elastic constants C_44_, C_11_ & C_11_ + C_22_ for (**a**) AlCoCuFeNi (**b**) AlCoCrFeNi and (**c**) AlCoCrFeNiTi.

The role of Mn on the driving force has also been illustrated (see Figure A4), where addition of Mn within 0.05 mole fraction leads to a higher entropy contribution than the enthalpy term, but an addition beyond 0.05 mole fraction increases the enthalpy substantially and leads to the formation of intermetallic compounds. The driving force required to form B2_BCC phase was not impacted significantly by the addition of Mn even when the temperature was varied. The role of Cu addition on the phase stabilities was also investigated, and the results are presented in Figure A5, which shows that although Cu does not affect the entropy of mixing, Cu addition increases the enthalpy of mixing as the temperature is increased. The driving force required is very high to incorporate Cu within the mole fraction <0.14 due to the high enthalpy of mixing in this range which tends to form intermetallic compounds. The driving force required to incorporate Cu mole fraction >0.16 is considerably less since the enthalpy of mixing is considerably lower in this case. For the density of states calculation, the authors used the PBE exchange-correlation, where charge density and kinetic energy threshold for the wave function, were kept constant to 850 Ry and 85 Ry, respectively. We have shown deformed HEA geometries and energy variations graphs for selected HEAs. Density functional theory (DFT) calculations (for elastic constants evaluation) were performed for more than 18 alloys, and associated results are shown in Table A3. We have shown deformed HEA geometries and energy variations graphs for selected HEAs (See Figure 4, Figure A6 and Figure A7).

### 3.2. Analysis of Elastic Properties and Shortlisting of HEAs

**Figure 5:**
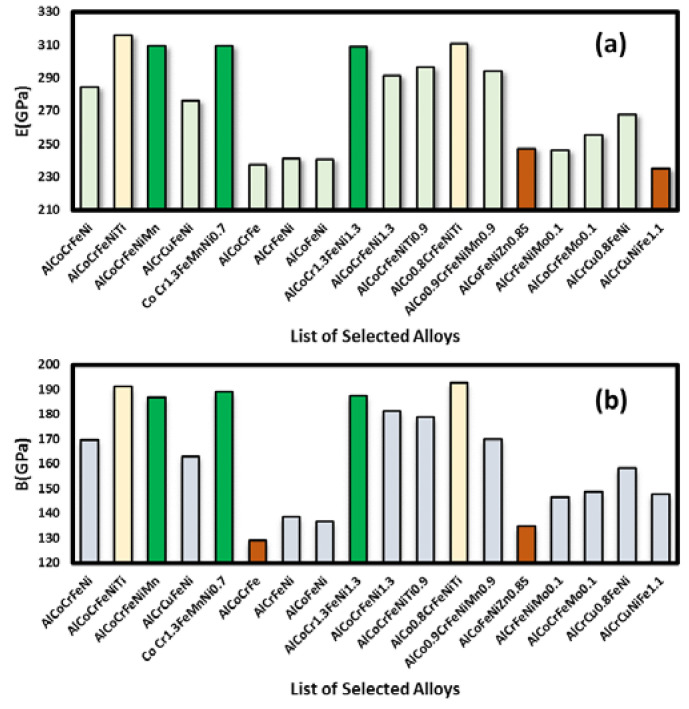
(**a**) Elastic constants (*E*) and (**b**) Bulk modulus (*B*) for selected alloys. Alloys shown in green bar are titanium free but elastic constants are not very different from titanium-based alloys (shown in yellow bar). Red bars are for low performance alloys.

**Figure A8:**
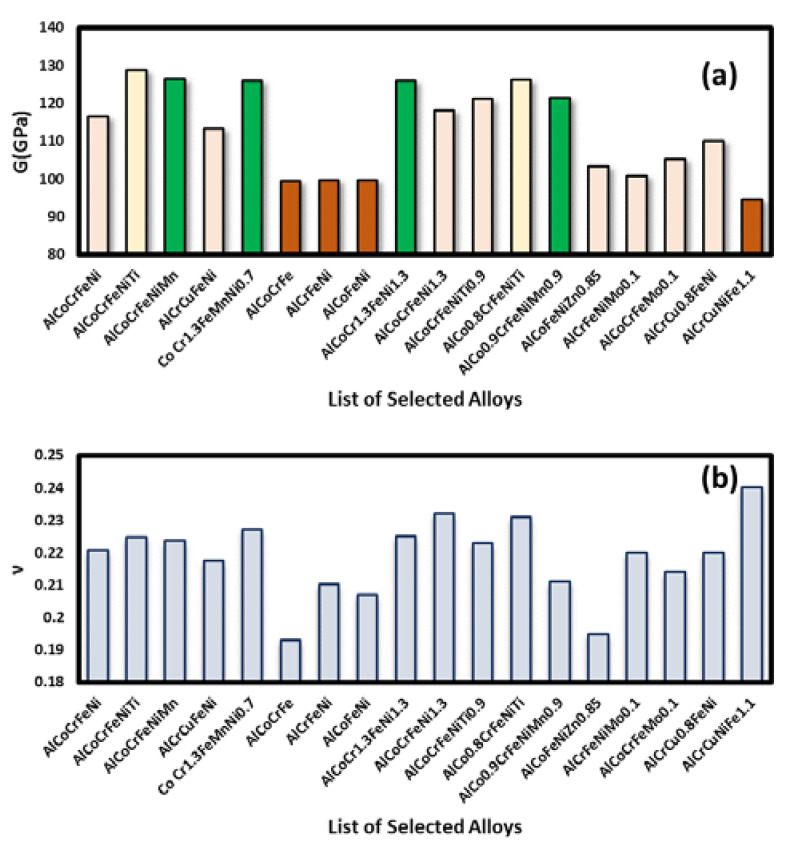
(**a**) Shear modulus (G) and (**b**) Poisson’s ratio (ν) for selected alloys. Alloys shown in green bar are titanium free but elastic constants are not very different from titanium-based alloys (shown in yellow bar). Red bars are for low performance alloys.

It can be seen that among the selected titanium-free alloys, AlCoCrFeNiMn, CoCr_1.3_FeMnNi_0.7_ and AlCoCrFeNi_1.3_ exhibited good elastic properties. All elastic constant values (*E, B*, and *G*) are not very different from those of a Ti-based alloy (AlCoCrFeNiTi). It was observed that the elastic constants for four elements alloys are lower than other multi-element alloys of five or more elements. This is primarily due to the known thermodynamic fact that the configurational entropy of a binary alloy is maximum when the elements are in equiatomic proportion and that the maximum configurational entropy in any system increases with increasing number of elements in the system. High mixing entropy has a profound effect on the constituent phases, kinetics of phase formation, lattice strain, and thus properties. The effect of Ni enrichment in the parent AlCoCrFeNi alloy was predicted and observed. Similarly, the effect of Cr enrichment was also useful as can be seen in the case of the AlCoCr_1.3_FeNi_1.3_ alloy. Other alloys containing Cu and Zn exhibited relatively low performance as can be seen in Figure 5. Poisson’s ratio (*ν*) is shown in Figure A8.

**Figure A9:**
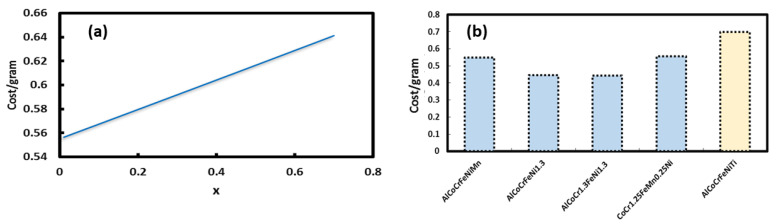
(**a**) Cost/gram for CoCr_1.3_FeMn_1+x_Ni_0.7−x_ alloy system for different values of ‘x’. It can be seen that cost increases linearly with respect to ‘x’. (**b**) Estimated cost/gram of shortlisted HEAs to be used for additive manufacturing.

Finally, it can be seen that there is a possibility to develop alternative titanium-free HEAs of comparable performance. Our initial assessment suggested that alloys such as AlCoCrFeNiMn, CoCr_1.3_FeMnNi_0.7_, AlCoCrFeNi_1.3_, and AlCoCr_1.3_FeNi_1.3_ should be further investigated. For one alloy, namely CoCr_1.3_FeMnNi_0.7_, we have modified the composition and observed that for the CoCr_1.3_FeMn_1+x_Ni_0.7−x_ alloy system, the lowest cost was for parent alloy: CoCr_1.3_FeMnNi_0.7_ (see Figure A9). A cost comparison was done for the four alloys as well as the Ti-based alloy AlCoCrFeNiTi. It can be seen that the estimated cost of these alloys will be less than Ti-based HEAs (see Figure A9). Hence, we have chosen four titanium free alloys as well as two Ti-based alloys namely AlCoCrFeNiTi and AlCo_0.8_CrFeNiTi for further processing. These shortlisted HEAs were prepared using elemental powders (purchased from Sigma Aldrich, St. Louis, MO, USA) and subsequent laser processing.

### 3.3. Density Calculations for Short-Listed Alloys

In order to calculate the density of each alloy, the following equation was used:

(25) $$\frac{1}{D_{HEA}}=\overset{n}{\underset{i=1}{\sum }}\frac{f_{i}}{D_{i}}$$

(26) $$f_{1}+f_{2\;}+f_{3}+\ldots +f_{n}=1$$

In other words, *D_HEA_* is the mean density of the mixture (or alloy), *D*_1_, *D*_2_, …, and *D_n_* are the densities of metal 1, metal 2, …, metal *n*, respectively. *f_i_* is the mass fraction of the respective element in the mixture. Table A4 shows the comparison of alloys and density values for the short-listed HEAs. It can be seen that density of most of the titanium-free shortlisted alloys is in the range of 6–7 g/cm^3^. The density of various other HEAs in the literature are also in a similar range [1,2,3,4,5,6,7,8,9,10]. An incorporation of aluminum in the alloy lowers its density, whereas enrichment of heavy density elements resulted in higher density as can be seen in the case of CoCr_1.3_FeMnNi_0.7_. The relative wear resistance of these materials should correlate with the bulk elastic modulus based on research in which a very good correlation was found for some alloy materials.

### 3.4. X-ray Diffraction Tests

**Figure 6:**
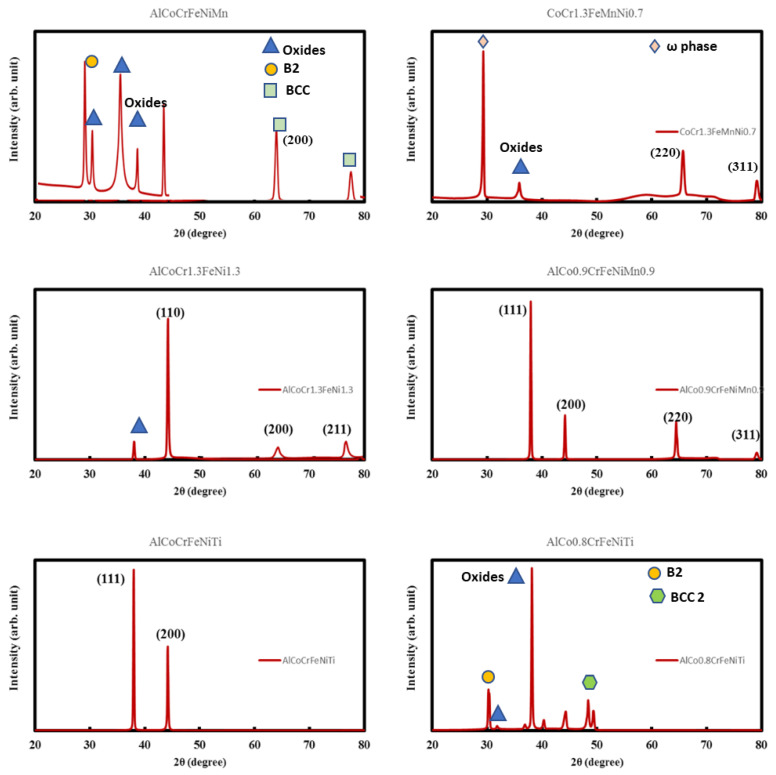
X-ray diffractograms of shortlisted HEAs. It can be seen that most of the alloys are free from secondary phases. We have observed both FCC and BCC lattice in these cases.

In order to characterize the phases and crystal structure of our alloy set, a Rigaku –Miniflex benchtop X-ray diffractometer (Rigaku, Wilmington, MA, USA) Cu Kα radiation was utilized. All additive manufactured samples were carefully removed from the steel substrate, in order to provide accurate information about their phase and purity. XRD measurement scans (see Figure 6) were carried out over the 2θ range of 20–80°.

The PDXL powder diffraction analysis software suite was used for analysis of the diffraction patterns and peaks [34,35]. High entropy alloys with the compositions AlCo_0.9_CrFeNiMn_0.9_ and AlCoCrFeNiTi were found to perfectly crystallize in an FCC lattice with their equilibrium lattice parameters (a0) matching well with earlier reported values. AlCoCrFeNiMn also exhibits peaks corresponding to a FCC lattice structure, though other peaks were also observed. This may be due to secondary phases. AlCoCr_1.3_FeNi_1.3_ also shows good crystallization and the three peaks (110), (200), and (211) shown in XRD are indicative of the BCC lattice. This was likewise seen as conforming with the way that FCC or BCC high entropy alloys of predominantly single phase are found broadly with the 3d transition elements combination as utilized in this investigation. A decent classical example in this angle is the notable HEA CrMnFeCoNi [36], which showed a high fracture toughness attributable to the event of glide dislocations bringing about improvement of cold working under mechanical deformation. In this manner, these three classes of HEAs could be viewed as promising possibilities for cryogenic applications attributable to the presence of the single phase, which advances nano-scale twinning. In some of our XRD patterns, secondary phases and oxides peaks have been noted (AlCoCrFeNiMn, CoCr_1.3_FeMnNi_0.7_, and AlCo_0.8_CrFeNiTi). Mostly oxide peaks, B2 phases, w phase, and BCC 2 phases have been noted, as indicated in the corresponding XRD patterns [37].

### 3.5. SEM/EBSD

**Figure 7:**
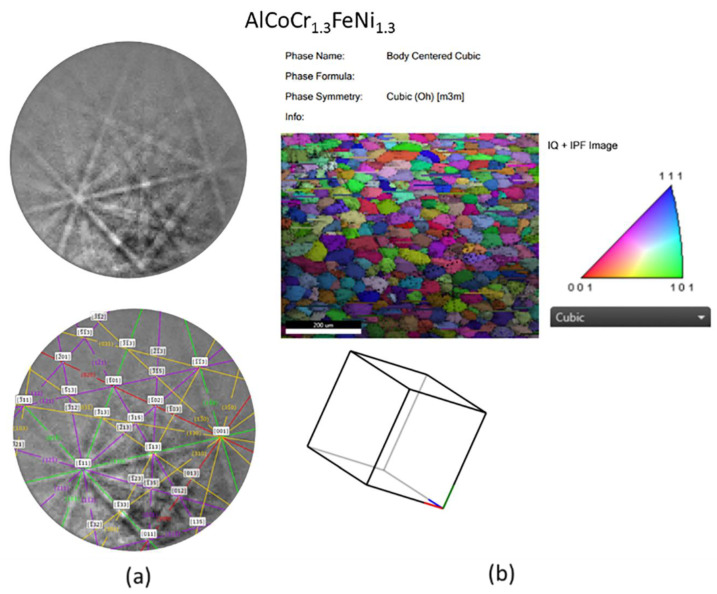
(**a**) Kikuchi pattern, and (**b**) IPF map and grain orientation distribution is shown for high entropy alloy AlCoCr_1.3_FeNi_1.3_. The alloy exhibited body centered cubic crystal structure as revealed in Kikuchi pattern and also XRD patterns also confirm the BCC type crystal structure.

**Figure A10:**
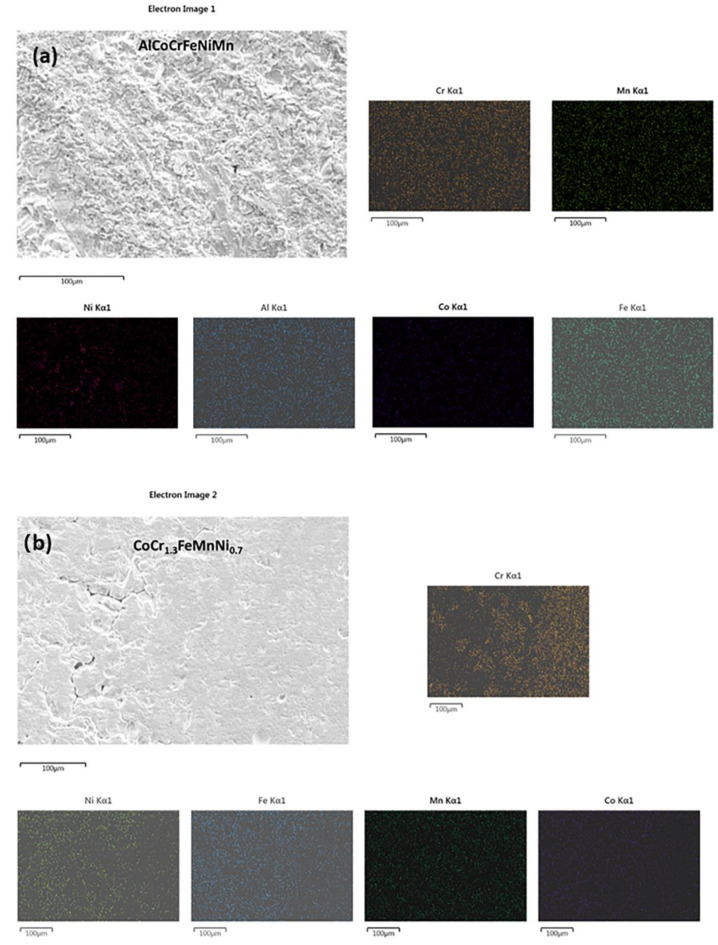
(**a**). SEM image and EDS elemental maps of additive manufactured HEA sample (AlCoCrFeNiMn). (**b**). SEM image and EDS elemental maps of additive manufactured HEA sample (CoCr_1.3_FeNi_0.7_Mn).

**Figure A11:**
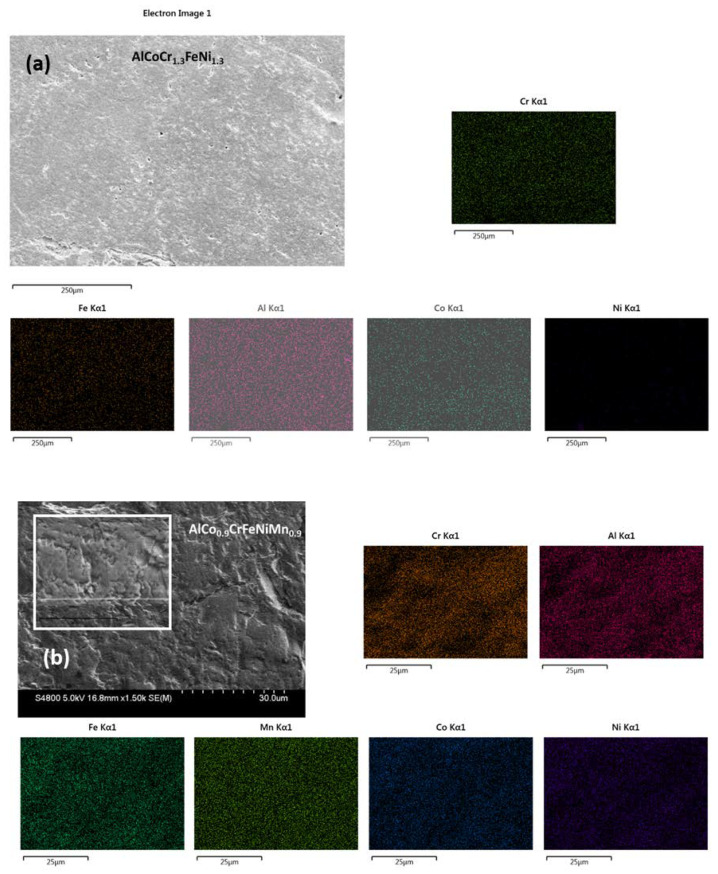
(**a**) SEM image and EDS elemental map of additive manufactured HEA sample (AlCoCr_1.3_FeNi_1.3_). (**b**). SEM image and EDS elemental map of additive manufactured HEA sample (AlCo_0.9_CrFeMn_0.9_Ni).

**Figure A12:**
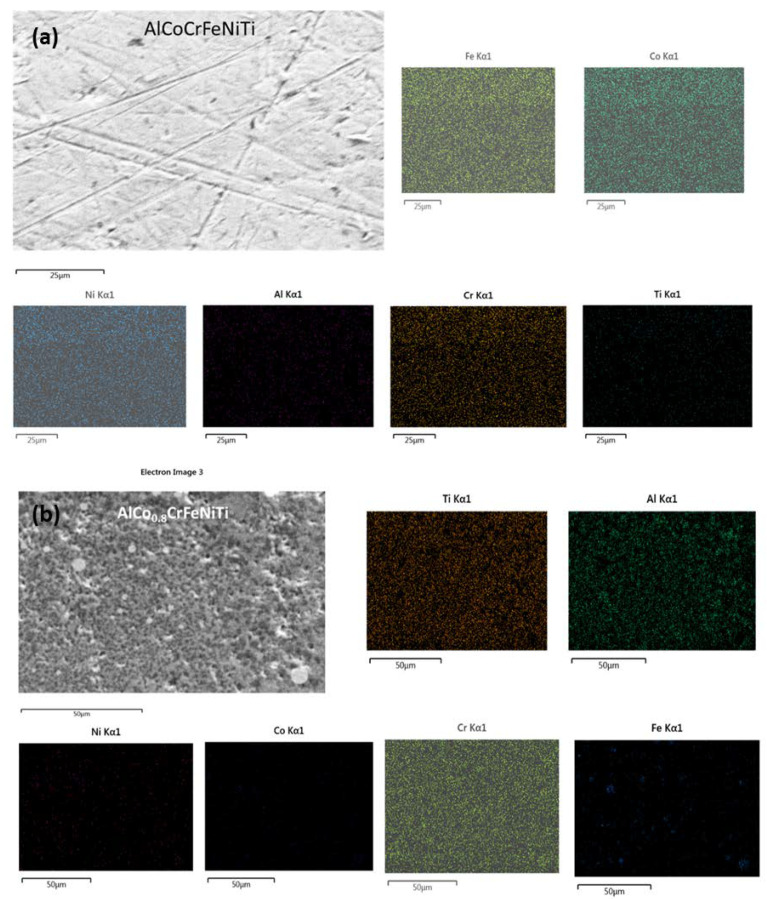
(**a**) SEM image and EDS elemental map of additive manufactured HEA sample (AlCoCrFeNiTi). (**b**) SEM image and EDS elemental map of additive manufactured HEA sample (AlCo_0.8_CrFeNiTi).

Figure A10, Figure A11 and Figure A12 show the microstructure and EDS elemental maps of HEA samples. EDS elemental maps indicate moreover a consistent elemental distribution with very little segregation or clustering in most cases. Scanning electron micrographs of different HEA samples are shown, and it can be seen that most of the samples appear free of secondary phases or precipitation. We have not seen any notable pores or continuous scan tracks in most of the samples. Most of these AM samples lack irregular and interrupted molten tracks as can be seen in images. The balling effect is also not prominent as evidenced from the surface morphology, thereby confirming a low tendency for a disorderly solidification front. For our set of measurements, the utilized high voltage in SEM imaging was 30 kV [38,39]. EDAX OIM software was utilized to analyze EBSD data (Figure 7). Strikingly, neither noticeable irregular bright white zones were spotted nor any dendritic secondary phases were realized in our samples. Such information reinforces the finding of single-phase alloy formation in a large portion of the introduced cases as affirmed by XRD.

### 3.6. Potentiodynamic Scans and EIS

**Figure 8:**
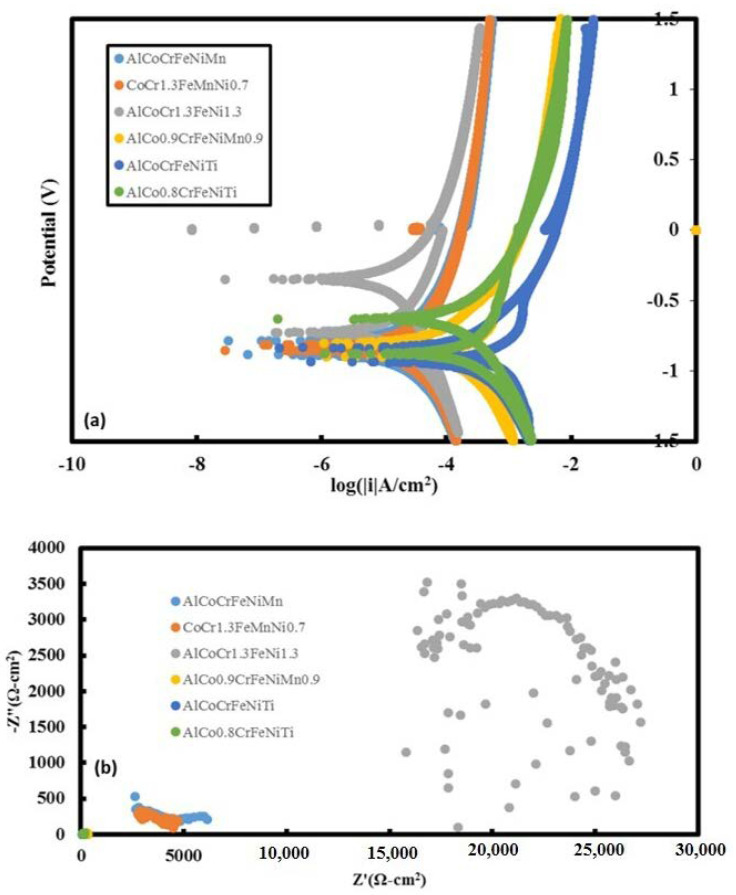
(**a**) Potentiodynamic scan plots (potential (*V* vs. *SCE*) vs. log |i|) for six different HEAs. (**b**) Nyquist plots for six different HEAs. It can be seen that AlCoCr_1.3_FeNi_1.3_ alloy performance is best performing alloy.

The potentiodynamic tests and associated results suggest that the titanium-free HEAs perform better than titanium-based alloys (see Figure 8 and Table 1). The EIS test results follow moreover a similar pattern, and it can be seen that AlCoCr_1.3_FeNi_1.3_ is the best performing alloy for both cases (corrosion current was ~10^−5.2^ (A/cm^2^)). We have compared the available data for pure titanium metal where electrochemical corrosion tests were conducted under a NaCl deposit in wet oxygen at 600 °C [40]. In that case the logarithm of corrosion current value was ~−4.35 (A/cm^2^) [40]. In the case of Ti-6Al-4V alloy the log (corrosion current) value in ~1% NaCl was ~−5.75 (A/cm^2^) [41]. In the case of γ Ti-Al alloy the corrosion current value was ~0.16 (μA/cm^2^). In previous report the details about the other corrosion tests were shown [41]. The corrosion tests (cyclic potentiodynamic polarizations) were performed using 3.5 wt.% NaCl solution in water [33]. These tests were performed based on the guidelines of ASTM G61 standards. All the polarization tests were carried out with reference to a saturated calomel electrode (SCE). The counter electrode used in the potentiodynamic polarization tests was platinum. The scan rate employed was 0.2 mV/s during the polarization test. Electrochemical impedance spectroscopy (EIS) tests were conducted in the potentiostatic mode with the following parameters: an AC signal amplitude of 5 mV, 5 points/decade and with a wide frequency range of 100 mHz–100 kHz. The potentiodynamic tests and associated results suggest that the titanium free HEAs are performing better than titanium-based alloys. The EIS test results follow a similar pattern, and it was noted that AlCoCr_1.3_FeNi_1.3_ is the best performing alloy for both cases (the logarithm of the corrosion current was ~−5.2 (A/cm^2^)). We have compared the available data for pure titanium metal where electrochemical corrosion tests were conducted under a NaCl deposit in wet oxygen at 600 °C [40]. In that case the logarithm of corrosion current value was ~−4.35 (A/cm^2^) [40]. In the case of Ti-6Al-4V alloy the log (corrosion current) value in ~1% NaCl was ~−5.75 (A/cm^2^) [41]. In case of γ Ti-Al alloy the corrosion current value was ~0.16 (μA/cm^2^). 

**Figure A13:**
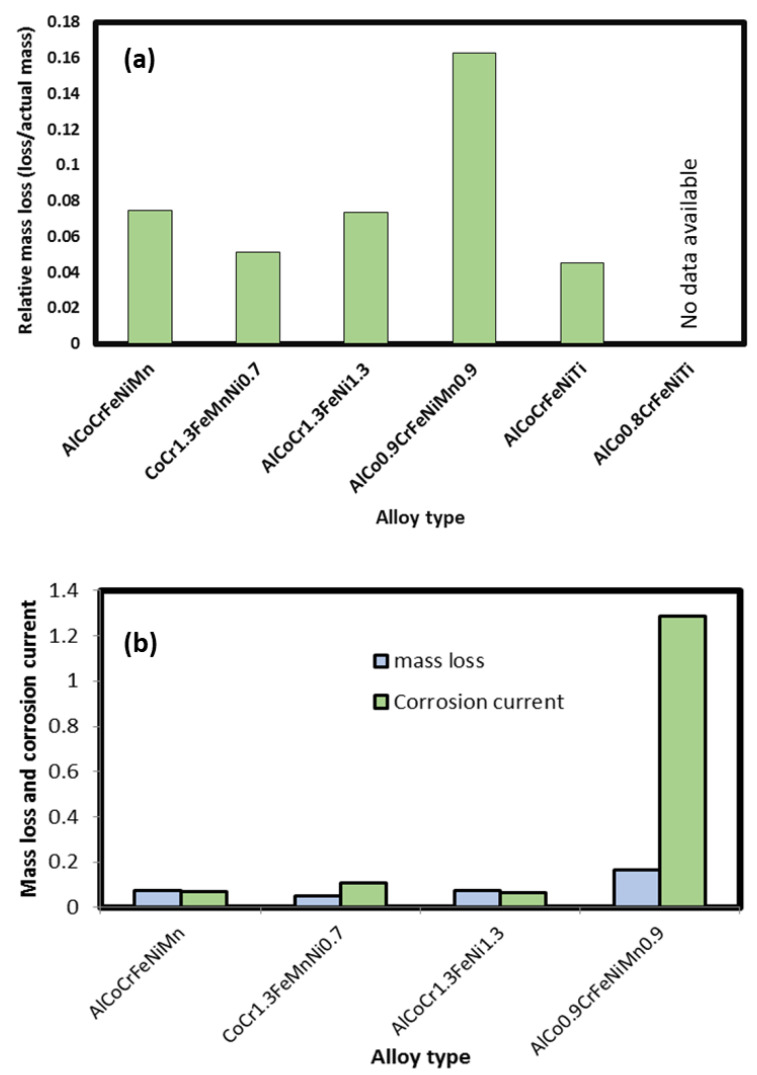
(**a**) Accelerated corrosion Mass loss test results for different HEAs. (**b**) The comparison of mass loss tests and corrosion current (as noted in potentiodynamic scans) for different HEAs.

In the case of accelerated corrosion-based mass loss tests (see Figure A13), the highest loss was noted for alloy 4, namely AlCo_0.9_CrFeNiMn_0.9_. Whereas, the lowest loss was noted for alloy 2 (CoCr_1.3_FeMnNi_0.7_). This is moreover consistent with potentiodynamic scan test results and associated corrosion currents.

**Figure 9:**
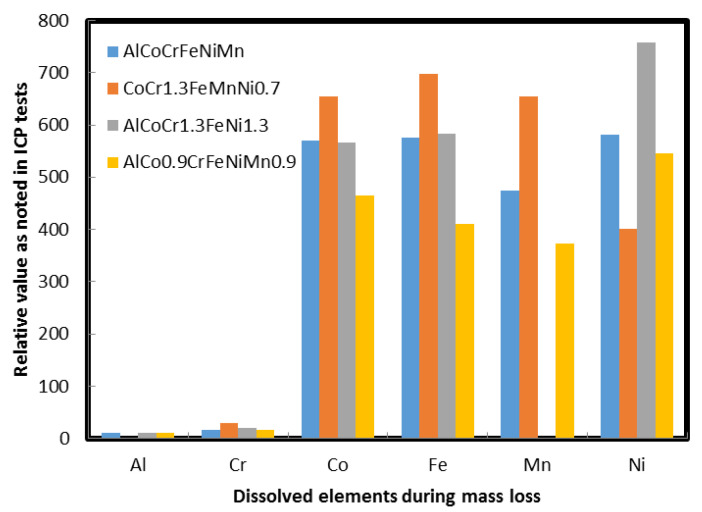
A bar chart showing the trend of elements that were dissolved (from HEAs) after mass loss tests (accelerated corrosion). The alloy showing null Mn concentration is Mn free alloy.

**Figure A14:**
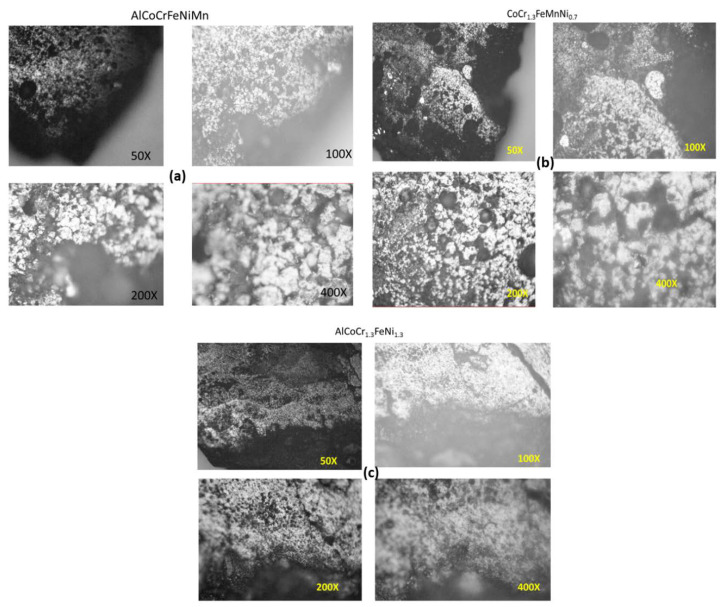
(**a**) The optical microscope images of alloy AlCoCrFeNiMn sample after 5 days of mass loss testing. (**b**) The optical microscope images of alloy CoCr_1.3_FeMnNi_0.7_ sample after 5 days of mass loss testing. (**c**) The optical microscope images of alloy AlCoCr_1.3_ FeNi_1.3_ sample after 5 days of mass loss testing.

We have also examined the HEA samples after 5 days of mass loss (Figure A13). Although no obvious cracks were noted, the surface of samples shows small pores throughout the samples, as shown in Figure A14. The solution retained after mass loss tests was also examined. For such chemical analysis inductively coupled plasma-mass spectroscopy analyses were performed. The purpose of these tests was to know more about the metal that is prone to dissolve. All analyses were performed in accordance to the NELAP protocols unless noted otherwise. The reporting limit must not be confused with any regulatory limit. Analytical results are reported to three significant figures for quality control and calculation purposes. It can be seen that for all cases aluminum and chromium were the most corrosion-resistant elements whereas Fe or Ni were the least corrosion resistant elements. A bar chart was prepared to compare these results (Figure 9). In our previous report [28] we have shown that Co was a dominant player with 1.134 wt.% loss in AlCoFeNiTiV_0.9_Sm_0.1_ compared to 0.896 wt.% in AlCoFeNiV_0.9_Sm_0.1_, which was consistent with the likelihood of Laves phase formation predicted by CALPHAD, and this contributed to additional corrosion for this alloy. The report also showed the lowest weight loss for the case of aluminum in those alloys.

### 3.7. Hardness Tests

**Figure A15:**
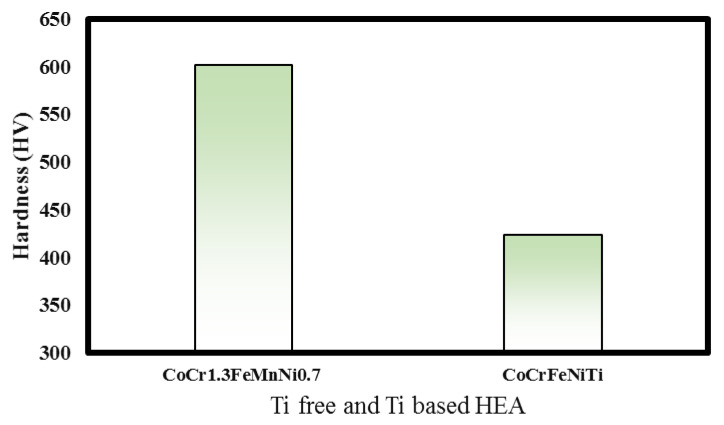
Hardness for best performing Ti-free and Ti based HEA sample.

We have performed hardnness testing (see Figure A15) and observed that the hardness value for CoCr_1.3_FeMnNi_0.7_ alloy was ~602 (HV). This hardness value was compared to other similar alloys (e.g., CoCrFeMnNi), and it was observed that the hardness value was low for CoCrFeMnNi ~176 [42]. The hardness value for AlCoCrFeNi alloy was ~400 [42]. No hardness data in the literature for the alloy AlCoCrFeNiMn and AlCoCrFeNiTi were found. The reported hardness value for AlCoCrFeNiTi_0.5_ was ~178 (HV), however in our case the hardness value was ~240 for AlCoCrFeNiTi. In the literature, the data for the Al_x_CoCrFeMnNi system suggests an increase in hardness when x changes from 0.10 to 1.25. We have also noted that in some cases Mo based alloys (Al_a_Co_b_CrFeMo_x_Ni_y_) exhibit high hardness (maximum ~801 HV for the case of AlCo_0.5_CrFeMo_0.5_Ni), however, we have noted the presence of intermetallic phases in this alloy series as well as reduced elastic constants (bulk and shear modulus) for these cases, as have been reported. A Ti-based sample was also tested and it was observed that the best performing single phase samples (CoCrFeNiTi) show a hardness value of ~424.16 HV, which is still less than the Ti-free sample’s hardness.

### 3.8. Factorial Design of Experiments Laser Printing Test Matrix

**Figure A16:**
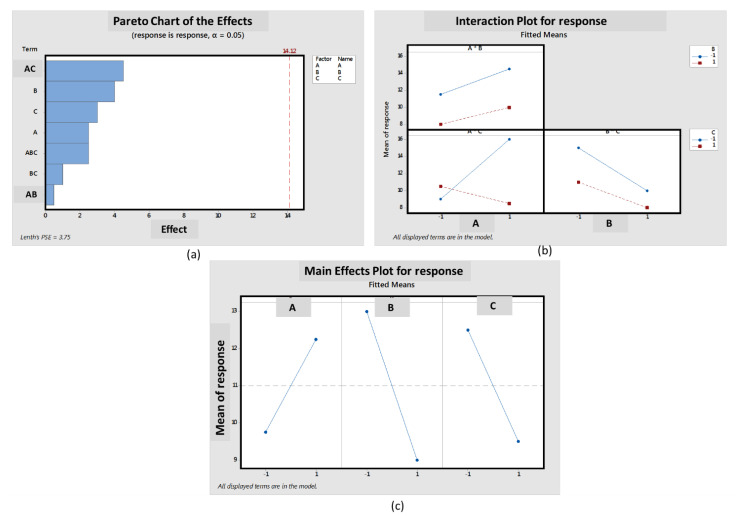
Analysis of the factorial design tests: (**a**) Pareto chart of the effect; (**b**) Interaction plot for response; and (**c**) main effects plot for response. It can be seen that high power and high scan speed is detrimental for PBF processing. (A = laser power, B = scan speed, C = substrate thickness).

**Figure A17:**
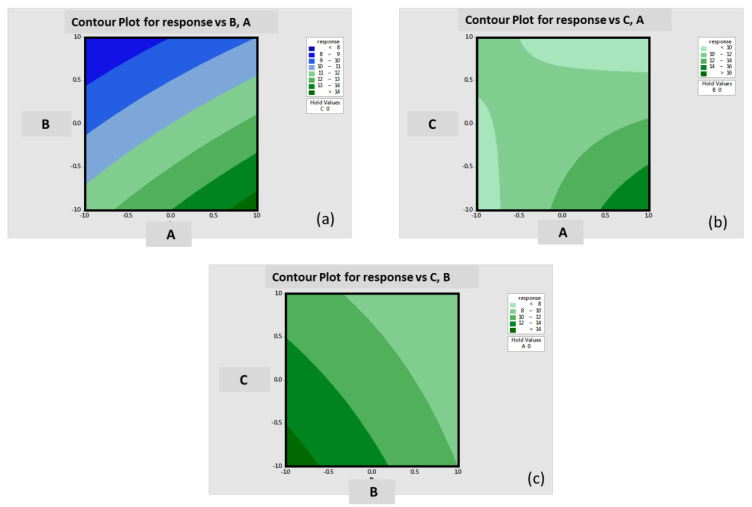
Contour plots for different variable vs. response (A = power, B = speed, C = thickness). (**a**). Plot for response vs B, A (**b**). Plot for response vs C, A (**c**). Plot for response vs C, B.

**Figure A18:**
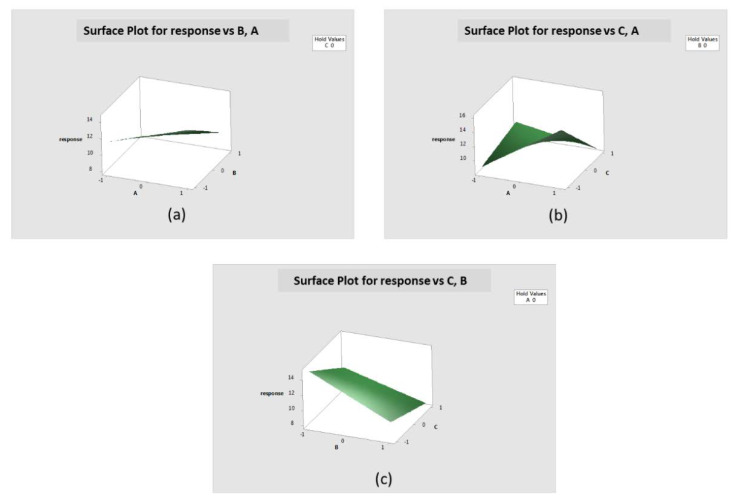
Surface plots for different variable vs. response (A = power, B = speed, C = thickness) (**a**). Plot for response vs B, A (**b**). Plot for response vs C, A (**c**). Plot for response vs C, B.

**Figure A19:**
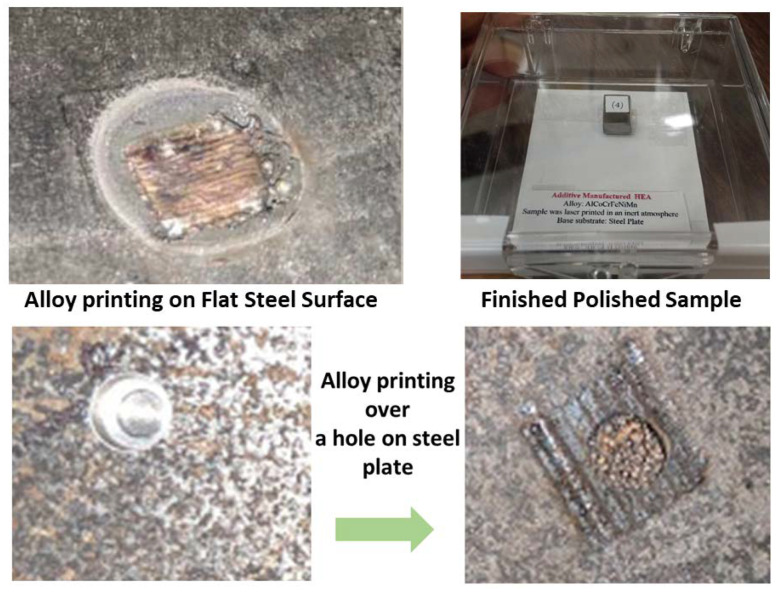
Alloy deposition (three layers) after optimization of various parameters.

**Figure A20:**
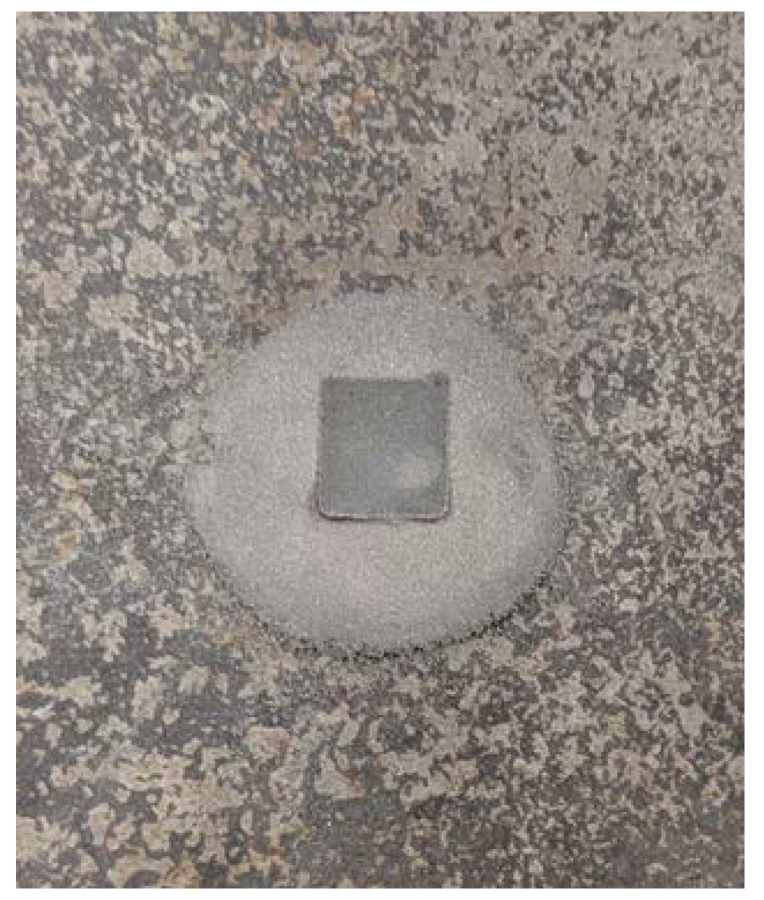
A square shaped HEA printing using selective laser melting technique.

After analysis of the data, different charts and graphs were created. Pareto charts of effects, interaction plot for response, main effect plots for response, and contour and surface charts of responses are shown in Figure A16, Figure A17 and Figure A18. It can be seen that the best combination was relatively low scan speed and relatively low substrate thickness. In the Pareto chart the highest effect was observed for AC combination, whereas the lowest effect was associated with the AB combination. It was also noted that in the case of low power and low thickness at the high scan speed, there was less deposition as shown in Table A5. Note that the results will vary from alloy to alloy. In the present case the results are shown for alloy 3 (AlCoCr_1.3_FeNi_1.3_). Samples are shown in Figure A19 and Figure A20. The choice of alloy 3 was based on its better XRD results and phase purity and due to time constraint, the design of experiment is not possible for all sets for alloy. This will be a future research topic of the PIs.

### 3.9. Effect of Temperature (X-ray Diffraction Tests on Arc Melted Samples)

**Figure A21:**
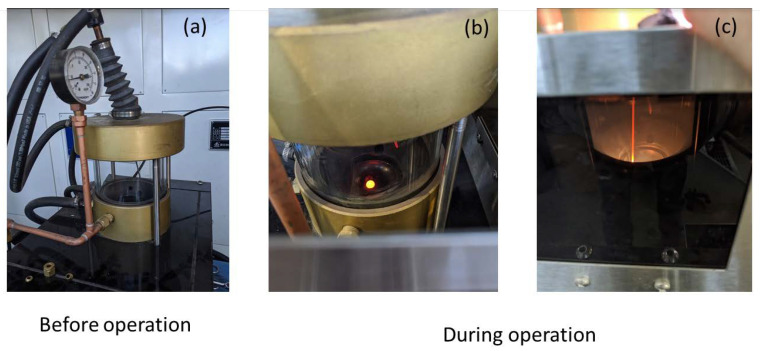
The digital images of an arc melting apparatus used for HEA fabrication (**a**). Electric Arc Melter (**b**). Ignition (**c**). Charging and Melting.

**Figure A22:**
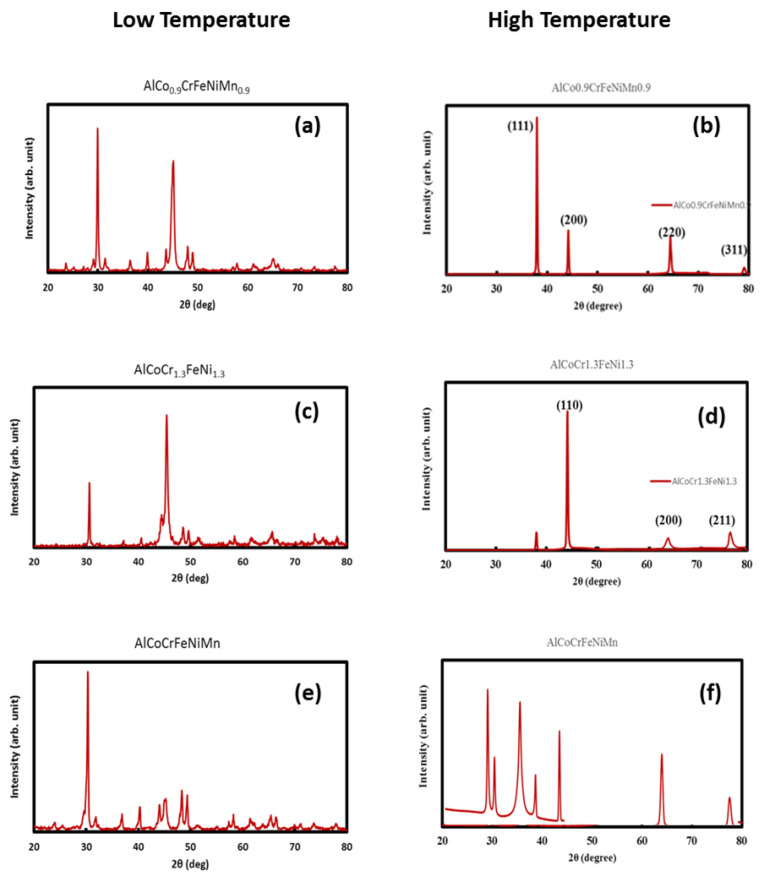
(**a**–**f**) X-ray diffractograms of shortlisted HEAs. It can be seen that most of the alloys fabricated at relatively low temperature have more secondary phases and impurity peaks. We have observed both FCC and BCC lattices in these cases.

All samples prepared at two different temperatures (~1800 °C and 1300 °C) were carefully removed from the steel substrate, in order to provide accurate information about their phase and purity. These samples were characterized using X-Ray diffractometry. The temperature value for the AM samples is within the typical temperature window for most additively manufactured alloys and also depends on factors such as input laser power, hatch spacing, material type, and exposure time [43,44]. The low temperature range value is close to that reported for a variety of titanium-free alloys [45] For low temperature tests, all six alloys were synthesized using arc-melting (Figure A21). This was also done in order to compare direct laser sintered alloys to the arc-melted samples. To prepare the samples, 5 g of well-mixed powder were placed into a Carver mechanical press inside of a die. The die was lubricated with a wax lube, which later burned off during arc-melting. The powder was pressed into pucks using 7 tons of pressure held for 30 s each. Puck diameter was 20 mm and a height of roughly 5 mm. The arc-melter used was a Gold Star 302 CC-DC Welding Power Source (Miller, Indianapolis, IN, USA) set to 200 max amps. Radnor tungsten electrodes that were 2% thoriated were ground to a point to create a better arc. Once the puck was placed inside the copper crucible, vacuum was pulled to −80 kPa, then back flowed with UHP argon 3 times. The first alloy tested was alloy 1, and the entire puck was placed inside the chamber. Once an arc was created, it became apparent that the sample was too large to properly melt. Due to this, this puck and each subsequent puck for all six alloys were broken in half, resulting in pucks that weighed roughly 2.5 g each. All six samples were then cut using a water cooled saw in order to prevent degradation of their microstructures.XRD measurement scans (Figure A22) indicated that high entropy alloys of the compositions AlCo_0.9_CrFeNiMn_0.9_ and AlCoCrFeNiTi were found to crystallize in an FCC lattice. 

### 3.10. Reduced Elastic Constants for HEAs

**Figure 10:**
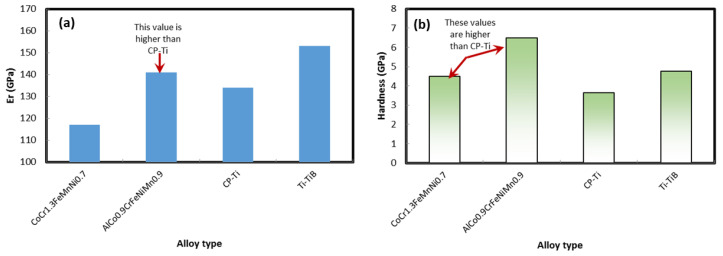
(**a**) A bar chart showing the reduced elastic constant for different HEAs as well as commercially pure Ti and Ti-TiB composite (PBF based). (**b**) A bar chart showing the hardness for different HEAs as well as commercially pure Ti and Ti-TiB composite (PBF based).

After hardness tests, we have examined and compared the reduced elastic constant *Er* as well as hardness of our newly designed alloys with Ti/Ti-B composite materials prepared using the selective laser melting technique [46]. For CP (commercially pure-Ti, maximum reported reduced elastic modulus was ~134 GPa (at load 2 mN); whereas a lower value ~102 GPa was obtained at an indentation load of ~5 mN. For the case of Ti-TiB composite maximum *Er* value ~153 GPa was noted for 2 mN indentation load whereas the reduced value of ~122 GPa was observed at an indentation load of ~10 mN [46]. In our case, we have observed the *E*_r_ value as high as ~141 GPa for alloy 4(AlCo_0.9_CrFeNiMn_0.3_). For alloy 2 (CoCr_1.3_FeMnNi_0.7_) this value was ~117 GPa, which is still higher than CP-Ti. Similarly, the nanohardness of the samples were examined and compared with existing Ti based alloys. For Pure CP-Ti maximum noted hardness was ~3.64 GPa (at load 2 mN) whereas a lower value ~2.39 GPa was observed at load of 10 mN. Similarly, a high hardness value ~4.75 GPa was noted for Ti-TiB composite. In another report PBF-processed Ti-13Nb-13Zr alloys [47] exhibited the maximum hardness ~6.25. However, alloy 4 (AlCo_0.9_CrFeNiMn_0.9_) exhibited hardness value of ~6.5 GPa. The alloy-2 exhibited a lower hardness value of ~4.5. However, rests of the alloys have shown hardness value greater than 5.0 GPa. The *H* and the *E*_r_ of the alloys were characterized as:

*H*=*F*_max_/A_c_ = *F*_max_/26.43*hc*^2^ (27)

where *F_max_* is the maximum indentation load, *h_c_* is the contact depth under the maximum indentation load. A bar chart showing the reduced elastic constant for different HEAs as well as commercially pure Ti and Ti-TiB composite (PBF-based) in Figure 10a,b showed the bar chart of the hardness for different HEAs as well as commercially pure Ti and Ti-TiB composite (PBF-based).

*E*_r_ = (1 − *v*_s_)/E_s_ + (1 − *v_i_^2^*)*E*_i_ (28)

where vs. and *v_i_* are the Poisson’s ratio of the sample and the indenter, respectively, while *E*_s_ and *E*_i_ are the Young’s modulus of the samples and the indenter.

## 4. Conclusions

Overall, it is fully evident that the newly designed high entropy alloys’ performance (as revealed in Er and Hardness tests) is equal or superior to that of a variety of PBF-processed Ti-based alloys or commercially pure-Ti as discussed in the literature. It was also noted that the Ti-Nb-Zr alloy reported in the literature is not superior to the new alloys reported in this study. However, more testing and evaluation will be needed to examine the long-term performance and repeatability. ASTM standard corrosion tests were performed to evaluate the performance of the HEAs. In the present piece of research we have presented details about the design, cost optimization of alloys, details about laser tests, methods to control the laser, optimization methods, design of experiments for evaluation of various parameters and their effects on the process, effect of temperature and method of fabrication, EBSD tests, accelerated corrosion tests, chemical analysis after corrosion testing, microstructural and EBSD analysis as well as mechanical properties of the HEAs. The HEAs show BCC or FCC type crystal structures that were validated in Kikuchi patterns. It is essential to remember that these alloys were first designed considering the lowest cost combination. Hence, higher performance can be expected for future tests.

## Figures and Tables

**Table 1:** List of shortlisted HEAs and associated potentiodynamic scan parameters for corrosion performance.

Additive Manufactured Alloy Stoichiometry	E_corr_ (V)	Log I_corr_ (A/cm^2^)
AlCoCrFeNiMn	−0.8838	−5.15
CoCr_1.3_FeMnNi_0.7_	−0.866	−4.97
AlCoCr_1.3_FeNi_1.3_	−0.724	−5.19
AlCo_0.9_CrFeNiMn_0.9_	−0.903	−3.89
AlCoCrFeNiTi	−0.933	−3.64
AlCo_0.8_CrFeNiTi	−0.874	−3.75

## Appendix A

**Table A1:** List of High Entropy Alloy Composition.

Sr. no	High Entropy Alloy Composition	Sr. no	High Entropy Alloy Composition
1	Al_0.125_CoCrCuFeMnNiTiV	41	Al_1.2_CrFe_1.5_MnNi_0.5_
2	Al_0.25_CoCrCuFeMnNiTiV	42	CoCrCuFeMnNi
3	Al_0.25_CoCrCu_0.75_FeNi	43	CoCrCu_0.5_FeNi
4	Al_0.3_CoCrCuFeNi	44	CoCrCuFeNi
5	Al_0.5_CoCrCu_0.5_FeNi	45	Co_0.25_Cr_0.25_FeMn
6	Al_0.5_CoCrCuFeNi	46	CoCr_0.4_Fe_8_Mn_5.4_Ni_5.2_
7	AlCoCrCu_0.25_FeNi	47	CoCr_0.75_FeMn_0.75_Ni
8	AlCo_3.5_CrCu_0.5_FeNi	48	CoCrFe_0.5_Mn_0.5_Ni_1.5_
9	Al_0.5_CoCrCuFeNiTi_0.2_	49	CoCrFeMnNi
10	Al_0.25_CoCrCuFeNiTiMnV	50	CoCr_1.25_FeMn_0.25_Ni
11	AlCoCrCu_0.5_Ni	51	CoCr_1.3_FeMnNi_0.7_
12	Al_0.02_CoCrFeMnNi	52	Co_1.4_CrFeMnNi
13	Al_0.03_CoCrFeMnNi	53	Co_1.5_Cr_0.5_FeMn_0.5_Ni
14	Al_0.04_CoCrFeMnNi	54	CoCrFeMo_0.3_Ni
15	Al_0.08_CoCrFeMnNi	55	Co_1.5_CrFeMo_0.1_Ni_1.5_Ti_0.5_
16	AlCo_0.5_Cr_0.5_Fe_0.5_MnNiV_0.5_	56	CoCrFeNi
17	AlCo_0.5_Cr_0.5_Fe_0.5_MnNiV	57	Co_1.5_CrFeNi_1.5_Ti_0.5_
18	Al_0.3_CoCrFeMo_0.1_Ni	58	CoCrMnNi
19	AlCoCrFeMo_0.1_Ni	59	CoCuFeMnNiSn_0.03_
20	Al_0.1_CoCrFeNi	60	CoCuFeNi
21	Al_0.25_CoCrFeNi	61	CoCuFeNiTi
22	Al_0.375_CoCrFeNi	62	CoFeMnNi
23	Al_0.4_CoCrFeNi	63	CrCuFeMn_2_Ni_2_
24	AlCoCrFeNi	64	Cr_0.66_FeMnNi
25	AlCoCrFeNiSi_0.2_	65	CoFeNiSi_0.25_
26	Al_0.2_Co_1.5_CrFeNi1.5Ti_0.5_	66	CoMnNi
27	Al_0.3_CoCrFeNiTi_0.1_	67	CrFeNi
28	AlCo_3_CuFeNi	–	–
29	Al_0.25_CoFeNi	–	–
30	AlCoFeNiTi	–	–
31	Al_0.2_CrCuFeNi	–	–
32	Al_0.2_CrCuFeNi_2_	–	–
33	Al_0.4_CrCuFeNi	–	–
34	Al_0.4_CrCuFeNi_2_	–	–
35	Al_0.5_CrCuFeNi	–	–
36	Al_0.6_CrCuFeNi_2_	–	–
37	Al_0.7_CrCuFeNi	–	–
38	Al_0.8_CrCuFeNi_2_	–	–
39	Al_0.5_CrFe_1.5_MnNi_0.5_	–	–
40	Al_0.8_CrFe_1.5_MnNi_0.5_	–	–

**Table A2:** 3 Factor design of experiments at high and low levels.

Run	A	B	C	AB	AC	BC	ABC	Observation
1	−	−	−	+	+	+	−	Y_1_
2	+	−	−	−	−	+	+	Y_2_
3	−	+	−	−	+	−	+	Y_3_
4	+	+	−	+	−	−	+	Y_4_
5	−	−	+	+	−	−	+	Y_5_
6	+	−	+	−	+	−	−	Y_6_
7	−	+	+	−	−	+	−	Y_7_
8	+	+	+	+	+	+	+	Y_8_

**Table A3:** DFT calculated three independent elastic constants (C_ij_), elastic (E), bulk (B), shear modulus (G), Poisson’s ratio (ν) & equilibrium lattice distance (a_0_) for the following HEAs.

High Entropy Alloy Composition	C_11_(GPa)	C_12_(GPa)	C_44_(GPa)	E(GPa)	B(GPa)	G(GPa)	B/G	ν	a_0_(Å)
AlCoCrFeNi	214.82	135.38	167.59	284.31	169.74	116.442	1.45	0.2208	3.567
AlCoCrFeNiTi	253.69	157.39	182.67	315.67	191.23	128.862	1.48	0.2248	3.781
AlCoCrFeNiMn	248.71	153.61	179.14	309.60	186.77	126.504	1.47	0.2237	3.763
AlCrCuFeNi	210.37	126.94	161.09	275.99	162.87	113.34	1.43	0.2175	3.579
CoCr_1.3_FeMnNi_0.7_	251.97	155.83	177.91	309.19	188.93	125.974	1.49	0.2272	3.774
AlCoCrFe	168.94	84.96	137.93	237.53	128.96	99.554	1.29	0.1930	3.462
AlCrFeNi	176.58	97.89	139.79	241.11	138.69	99.612	1.39	0.2102	3.458
AlCoFeNi	173.71	92.68	138.95	240.37	136.74	99.576	1.37	0.2070	3.467
AlCoCr_1.3_FeNi_1.3_	262.82	151.73	172.98	308.79	187.38	126.01	1.49	0.225	4.091
AlCoCrFeNi_1.3_	237.83	134.97	162.67	291.22	181.23	118.17	1.53	0.232	3.994
AlCoCrFeNiTi_0.9_	257.99	144.58	164.14	296.51	178.77	121.17	1.47	0.223	4.203
AlCo_0.8_CrFeNiTi	260.96	118.74	162.96	310.85	192.87	126.22	1.53	0.231	4.116
AlCo_0.9_CrFeNiMn_0.9_	242.66	142.68	168.93	294.03	169.85	121.35	1.4	0.211	4.118
AlCoFeNiZn_0.85_	181.83	102.75	145.89	247.01	134.98	103.35	1.31	0.195	3.962
AlCrFeNiMo_0.1_	185.58	112.89	143.96	246.27	146.69	100.91	1.45	0.220	3.467
AlCoCrFeMo_0.1_	188.71	117.68	151.79	255.55	148.74	105.28	1.41	0.214	3.463
AlCrCu_0.8_FeNi	201.2	131.1	160.2	267.84	158.1	110.14	1.44	0.22	3.98
AlCrCuNiFe_1.1_	170.1	95.7	133.4	235.2	147.7	94.66	1.56	0.24	3.65

**Table A4:** Comparison of alloys and density values for short-listed HEAs.

Alloy	Density (g/cm^3^)
AlCoCrFeNiMn	6.79
CoCr_1.3_FeMnNi_0.7_	7.86
AlCoCrFeNi_1.3_	6.84
AlCoCr_1.3_FeNi_1.3_	6.86275
AlCoCrFeNiTi	6.22
AlCo_0.9_CrFeNiMn_0.9_	6.75

**Table A5:** Factorial design of experiments laser printing test matrix output.

Test ID	Run	Power Ratio A	Scan Speed Ratio B	Thickness C	(Approximate % of Powder Printed)
P25Speed003T2	1	−	−	−	10
P30Speed003T2	2	+	−	−	20
P25Speed005T2	3	−	+	−	8
P30Speed005T2	4	+	+	−	12
P25Speed003T4	5	−	−	+	13
P30Speed003T4	6	+	−	+	9
P25Speed005T4	7	−	+	+	8
P30Speed005T4	8	+	+	+	8
